# Genomic basis of homoploid hybrid speciation within chestnut trees

**DOI:** 10.1038/s41467-020-17111-w

**Published:** 2020-07-06

**Authors:** Yongshuai Sun, Zhiqiang Lu, Xingfu Zhu, Hui Ma

**Affiliations:** 1 CAS Key Laboratory of Tropical Forest Ecology, Xishuangbanna Tropical Botanical Garden, Chinese Academy of Sciences, Mengla, 666303 Yunnan China; 2 Center for Plant Ecology, Core Botanical Gardens, Chinese Academy of Sciences, Mengla, 666303 Yunnan China

**Keywords:** Speciation, Genetic hybridization, Genomics, Plant hybridization

## Abstract

Hybridization can drive speciation. We examine the hypothesis that *Castanea henryi* var. *omeiensis* is an evolutionary lineage that originated from hybridization between two near-sympatric diploid taxa, *C. henryi* var*. henryi* and *C. mollissima*. We produce a high-quality genome assembly for *mollissima* and characterize evolutionary relationships among related chestnut taxa. Our results show that *C. henryi* var. *omeiensis* has a mosaic genome but has accumulated divergence in all 12 chromosomes. We observe positive correlation between admixture proportions and recombination rates across the genome. Candidate barrier genomic regions, which isolate var*. henryi* and *mollissima*, are re-assorted in the hybrid lineage. We further find that the putative barrier segments concentrate in genomic regions with less recombination, suggesting that interaction between natural selection and recombination shapes the evolution of hybrid genomes during hybrid speciation. This study highlights that reassortment of parental barriers is an important mechanism in generating biodiversity.

## Abstract (web summary)

Chinese chestnut is widely cultivated for nut production and harbors value as a genetic resource for restoration of American and European chestnut trees destroyed by chestnut blight. Here, the authors reveal the genomic basis of homoploid hybrid speciation within *Castanea* spp. and find the nonrandom distribution of reproductive barrier loci based on a high-quality reference genome.

## Introduction

Hybridization can generate species without changes in ploidy by reassembling genomes originating from different evolutionary histories into a lineage that is reproductively isolated from the parental lineages^1,2^. This mode of speciation, called homoploid hybrid speciation (HHS), is thought to occur only with difficulty because of, e.g., homogenizing effects from parental lineages^2^. However, theoretical and simulation-based studies suggest that HHS can occur through a reorganization of the preexisting variants which restrict gene exchange between parental lineages or establishing barriers by hybridization-induced chromosomal rearrangements^3–5^. Recently, the reassortment of parental barriers with or without fitness advantages has been considered to be likely as a common mechanism during hybrid speciation^6–9^. Particularly when meeting ecological opportunities, hybrid progenies with recombinant alleles may evolve to become an independent species under the influence of natural selection^6^. Unfortunately, it is still challenging to determine the genes underlying reproductive isolation (RI) between putatively parental species, hindering establishment of the causal link between hybridization and speciation^10^. Large numbers of artificial hybrids/crosses are necessary to locate the loci or genomic regions, which prevent gene flow between parental lineages using traditional approaches^7,9^, such as QTL or genetic mapping^11^. Thus more approaches are needed in order to understand the difference between model expectations and empirical findings for the incidence of HHS. Recent population genomic methods may provide opportunities to address this challenge.

Reduced introgression and enhanced divergence are expected to be exhibited at loci responsible for barriers to gene flow (i.e., barrier loci^12^) and the neutral loci linked to such barrier loci, due to selection against unfit hybrids. Genomic regions that are statistical outliers for divergence among populations have been a focus of attention in the recent past, with the aim of identifying barrier loci^12^. However, besides reproductive barriers between species, intraspecific factors (e.g., linked selection and demography) could also influence the genomic landscape of divergence^12^. Another approach for identifying barrier loci is the well-known genome cline analysis method, which compares locus-specific introgression with the genome-wide average, based on the fact that introgression can vary across the genome due to recombination^13^. A third approach is genomic scanning of linkage disequilibrium (LD) between unlinked genomic regions in hybrids, with the assumption that genetic incompatibilities separating species should show strong patterns of LD^14^. The above methods appear to be powerful in certain scenarios. However it is still unclear how heterogeneous recombination rates across genome affect these analyses. Here, we assumed that most genomic regions with mixed genetic compositions are likely to exert no/weak isolating effects and used a sophisticated experimental design and advanced approaches to capture historical strengthening of barrier effects and identify barrier genomic regions among chestnut lineages.

**Fig. 1:**
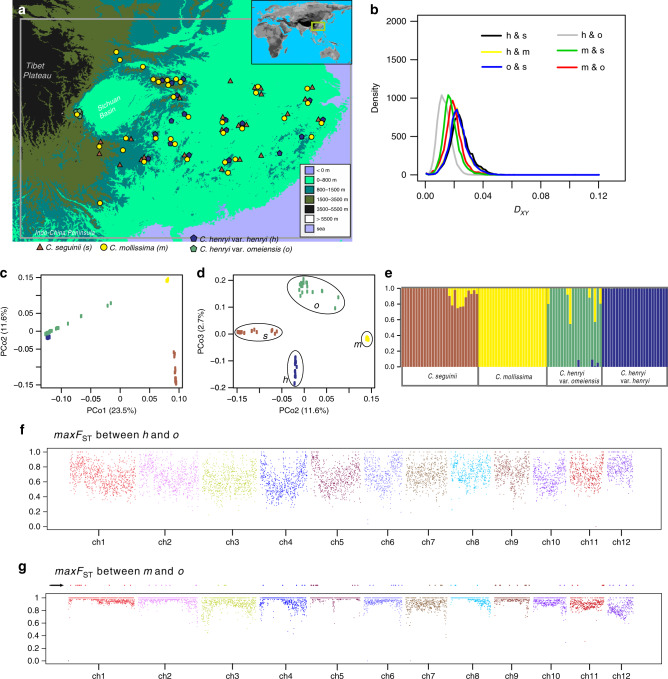
The four *Castanea* taxa show distinct genomic variation. **a** Map showing the distributions of samples collected. Each of the four taxa is shown in a different color. **b** Frequency polygon of *D*_XY_ per base pair for each pair out of the four taxa, using a sliding-window approach with a window size of 100 kbp. Abbreviations are: s = *C. seguinii*, m = *C. mollissima*, h = *C. henryi* var*. henryi*, o = *C. henryi* var*. omeiensis* (the same below). **c**, **d** PCoA showing the first three coordinates and the percentage of variation explained by each coordinate. The four colors represent samples collected from the four taxa. **e** Population structure estimated by admixture analysis for all four *Castanea* taxa. **f** *maxF*_ST_ analysis of 100 kbp nonoverlapping windows across the 12 chromosomes between *C. henryi* var*. henryi* and *C. henryi* var*. omeiensis*. The 12 chromosomes are represented by different colors. **g** *maxF*_ST_ analysis of 100-kb nonoverlapping windows across the 12 chromosomes between *C. mollissima* and *C. henryi* var*. omeiensis*. The arrow in **g** indicates windows where fixed differences between *C. henryi* var*. omeiensis* and the two parental taxa (*C. henryi* var*. henryi* and *C. mollissima*) can be observed. Source data underlying Fig. 1b–g are provided as a Source data file.

Chinese chestnut trees are thought to be critical for resurrecting wild American chestnut and European chestnut trees, which are being eliminated by *Cryphonectria parasitica*^15–18^. Knowledge of speciation genomics may be of great value to the chestnut forest restoration in Europe and America. We used phylogenetic control, the exclusiveness of barrier alleles in the hybrid lineage and backward simulations to identify/test candidate genomic regions responsible for RI between *Castanea mollissima* Blume^19^ and variety *henryi* of *Castanea henryi* (Skan) Rehder & E. H. Wilson. The candidate RI genomic regions would provide information for testing the role of hybridization in the origin of *C. henryi* var. *omeiensis*, which was discovered in the west of China (on Mount Emei) in 1964. Initially, it was suspected to be a hybrid lineage or hybrid (*C. mollissima* × *C. henryi* var*. henryi*). Morphologically, *C. mollissima* trees are characterized by three nuts per bur, differing from *C. henryi* trees which have one nut per bur^19^. However, the leaves of *C. henryi* var. *omeiensis* are covered with stellate tomentose hairs abaxially^19^, and morphologically similar to those of *C. mollissima*, providing inconclusive evidence for a hybrid origin hypothesis. Geographically^20^, *C. henryi*, endemic in China, appears to be broadly sympatric across most of the range of *C. mollissima* (Fig. 1a). The shrub-like species *Castanea seguinii*, characterized by glabrous leaves and three nuts per bur, also has a similar distributional range to *C. mollissima* and *C. henryi*. In addition, the flowering periods of *C. mollissima*, *C. seguinii* and *C. henryi* have overlap^19^, although some *C. mollissima* trees begin to flower in late April. Their distributions and flowering periods provide sufficient opportunities for interbreeding. Artificial hybridization experiments with *C. mollissima* and *C. henryi* showed that the seeding rate and empty-bur rate resulting from interbreeding were higher than those following normal pollination^21^. These interbreeding experiments and the distinct phenotypes of *C. mollissima* and *C. henryi*, together with their distributions, are suggestive of the existence of reproductive barriers typically isolating *C. mollissima* from *C. henryi*. A phylogenetic tree of all *Castanea* species suggested that these four taxa, which are endemic in China, constitute a monophyletic group, sister to the clade containing the European chestnut and the American chestnut^22^. Genetic variation within species indicated that *C. seguinii* and *C. mollissima* likely constituted a single clade although past hybridization might have occurred among these four diploid chestnut taxa^23^.

Here, we test the hypothesis that *C. henryi* var. *omeiensis* originated from hybridization between *C. henryi* var. *henryi* and *C. mollissima*, using multiple population genomic approaches and simulation-based tests. We identify candidate barrier genomic regions between *C. henryi* var. *henryi* and *C. mollissima*, based on genomic variation and a de novo assembled reference genome generated from Nanopore sequencing data^24^ and high-throughput chromosome conformation capture (Hi-C)^25^ short reads. We find that *C. henryi* var. *omeiensis* has a distinct genetic structure, and that both of the parental lineages contributed substantial genomic material to the hybrid lineage, a phenomenon referred to as genomic mosaicism^10^. Next, taking advantage of the phylogenetic control in this system, we identify the candidate barrier genomic regions by seeking genomic regions with reduced gene flow between parental lineages and filtering regions with mixed alleles from different species. Finally, we examine the mosaic of barrier genomic regions within the putatively hybrid lineage and investigate their distribution across chromosomes.

## Results

### Genome assembly

We chose one *C. mollissima* tree to perform single-molecule Nanopore DNA sequencing, and construct a Hi-C library for scaffolding after assembling reads into contigs; we selected this species because *C. mollissima* trees are cultivated widely in China and thus may have lower heterozygosity relative to the other three taxa (*C. seguinii*, *C. henryi* var. *henryi* and var. *omeiensis*). The haploid genome size of *C. mollissima* was measured by flow cytometry as 773–785 megabase pairs (Mbp), consistent with a previous report^26^. The Illumina HiSeq platform produced short reads with a total size of 80.8 billion base pairs (Gbp). The *k*-mer frequency analysis (*k* = 17) using Jellyfish^27^ reported a smaller genome size, of 706.3 Mb, relative to precious reports^26^. A total of 100.6 Gbp (~128× coverage) of Nanopore sequence data were used, after filtration, in the assembly analysis which produced 422 contigs with an *N*_50_ of 5.88 Mbp and total length of 773.99 Mbp (Supplementary Table 1). The longest contig was 41.15 Mb. We used Illumina short reads to polish these assembled contigs. Most of the Illumina short reads (98.45%) could be aligned to these 422 contigs using BWA-MEM^28^. We performed BUSCO V3.0.1^29^ assessment, and 97.4% of the core genes were found to be complete in the assembled genome. This assembly appears to be better than the previous version^26,30^, despite the smaller total genome size (Supplementary Table 1). Chromosome-scale scaffolding analysis based on Hi-C data assembled 377 contigs into 12 pseudo-chromosomes with a total length of 734.13 Mbp (Supplementary Fig. 1). More than half of the *C. mollissima* genome (59%) was identified as repetitive elements and 31.70% of the genome as retroelements (Supplementary Tables 2 and 3). 33.69% and 50.82% of the retroelements were classified as *Copia* and *Gypsy*, respectively. A total of 45,661 gene models were predicted, of which 45011 were protein coding genes (Supplementary Tables 4 and 5). This is considerably more than the 30832 gene models reported in the previous study^30^, needing further experimental confirmation.

### Population re-sequencing

We carried out whole-genome resequencing of 20 trees of *C. henryi* var. *omeiensis*, 24 *C. henryi* var. *henryi* trees, 43 *C. mollissima* trees and 28 *C. seguinii* trees, spanning their geographic ranges (Fig. 1; Supplementary Tables 6–9). We generated approximately 25.7× sequence coverage per individual tree by sequencing 2 × 150 bp paired-end reads. Reads were mapped on the high-quality de novo assembled reference genome created for *C. mollissima*. Then we identified heterozygous and homozygous genotype per base for each individual tree using a variant-calling pipeline and GATK4^31^. We validated the quality of the variant-calling procedure based on duplicated re-sequencing of seven trees. The maximum proportion of unverified heterozygotic genotypes for the seven trees was 0.000167%, corresponding to a Phred-scaled score of 57.8, suggesting that variants detected by our GATK4-pipeline were of high quality. The putative hybrid taxon *C. henryi* var. *omeiensis* had the highest observed heterozygosity (1.05 ± 0.26%), when compared to *C. mollissima* (0.66 ± 0.05%), *C. henryi* var. *henryi* (0.85 ± 0.08%) or *C. seguinii* (0.88 ± 0.11%). Sliding-window analysis with a size of 100 kilobase pairs (kbp) for each of the four taxa suggested that (Supplementary Fig. 2) *C. mollissima* had the lowest nucleotide diversity (*π* = 0.0075 ± 0.0039), compared to *C. henryi* (for var. *henryi*, 0.0108 ± 0.0045; for var. *omeiensis*, 0.0108 ± 0.0037) and *C. seguinii* (0.0100 ± 0.0043). The sliding-window analysis of mean nucleotide differences between lineages (*D*_XY_; 100 kbp) showed that the *D*_XY_ between *C. mollissima* and *C. seguinii* was less than the *D*_XY_ between *C. henryi* varieties (Fig. 1b), suggesting that divergence between *C. mollissima* and *C. seguinii* likely predated the origin of *C. henryi* var. *omeiensis*.

### Distinct genetic structure and fixed differences

Genetic analyses of genome-wide SNPs suggest that *C. henryi* var. *omeiensis* is an independent evolutionary lineage. First, a principal coordinate analysis (PCoA) indicated an intermediate position for *C. henryi* var. *omeiensis* along the second principal coordinate (PCoA2), but a distinct position for *C. henryi* var. *omeiensis* along PCoA3 (Fig. 1c, d). PCoA results were mirrored by the results of individual-based clustering analyses using ADMIXTURE^32^ and FASTSTRUCTURE^33^. For these four taxa, which had been identified based on morphological taxonomy, the best-fit model supported four distinct populations, albeit with substantial admixture among taxa (Fig. 1e). Eleven trees of *C. seguinii* seemed to be recent hybrids or backcrosses resulting from hybridization between *C. seguinii* and *C. mollissima*. With a setting of *K* = 5, FASTSTRUCTURE could not detect any extra distinct cluster. Finally, we used sliding-window analysis of fixation index (*F*_ST_, 100 kbp) to assess population differentiation between *C. henryi* var. *omeiensis* and its putative parental taxa, setting sample size per taxon to be 20. To investigate the fixed nucleotide difference, we used *maxF*_ST_, which represented the maximum *F*_ST_ per bp across one window. Consistent with observations of morphology^19^, windows with *maxF*_ST_ = 1.0 between *C. henryi* var. *henryi* and var. *omeiensis* were far fewer than those between *C. henryi* var. *omeiensis* and *C. mollissima* (Fig. 1f, g). Furthermore, this sliding-windows determination of *maxF*_ST_ showed that fixed differences between *C. henryi* var. *omeiensis* and the putative parental taxa could be found in any of the 12 chromosomes, especially at the chromosome peripheries. The high level of interspecific differentiation suggested that some reproductive barriers may have segregated *C. henryi* var. *omeiensis* from its parental lineages.

### Mosaic genomes

**Fig. 2:**
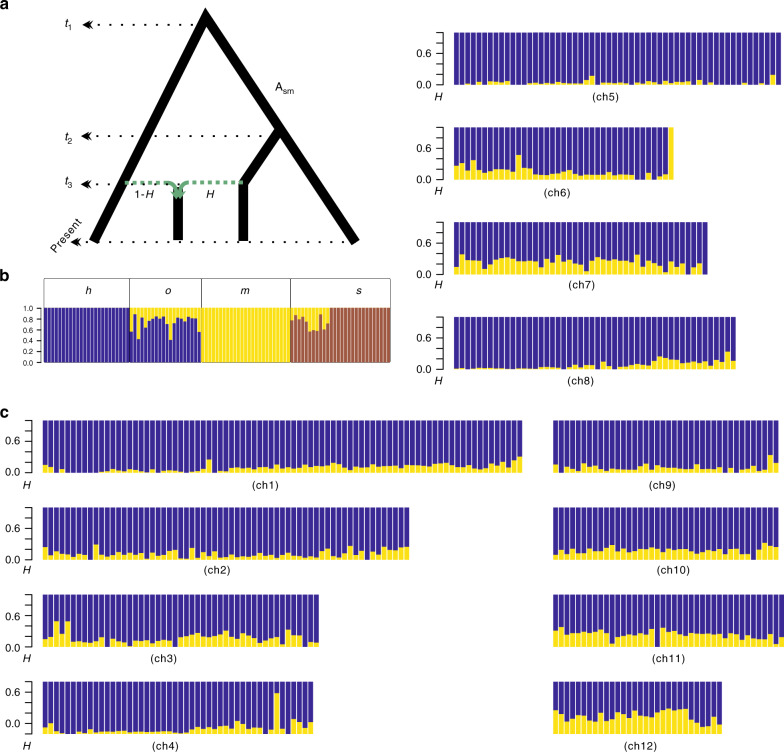
Genomic mosaicism. **a** The most likely evolutionary model used in the present study. At time *t*_1_, the lineage h diverged from the ancestral lineage A_sm_, which diverged into lineages s and m at time *t*_2_. At time *t*_3_, hybridization between m and h produced the hybrid lineage o. **b** Estimates of admixture proportions for each individual tree. **c** Estimated *H* for 100-kbp nonoverlapping windows across the 12 chromosomes of *C. henryi* var*. omeiensis*, from ch1 to ch12. Source data underlying (**b**, **c**) are provided as a Source Data file.

To investigate the genomic mosaicism, we introduced a statistic *hhs* = *d*_om_/*d*_hm_ × 100%, where *d*_om_ and *d*_hm_ represented mean nucleotide differences between *C. mollissima* and the two *C. henryi* varieties. If *C. mollissima* contributed some genetic components to the genome of *C. henryi* var*. omeiensis*, the statistic *hhs* would be <100%. The *hhs* estimated by our sliding-window (100 kbp) analysis was 86% on average and a bootstrap test significantly (*P* < 0.001) refuted the hypothesis of *hhs* = 100%. In concordance with this, coalescent modeling of the genome-wide interspecific site frequency spectrum (2D-SFS) generated by ANGSD^34^ clearly favored a hybrid speciation model leading to the formation of *C. henryi* var*. omeiensis* over models in which *C. henryi* var*. omeiensis* was sister to either *C. henryi* var*. henryi* or *C. mollissima* (Supplementary Fig. 3). In the hybrid speciation model, the *H* parameter represented the relative proportion of the genetic composition derived from *C. mollissima* (Fig. 2a). The estimated *H* was 24.4 ± 1.5%, indicative of *C. mollissima* being the minor parental taxon. To investigate in detail the parental contributions along each of the chromosomes, we split the genome into nonoverlapping windows (1 Mbp; Fig. 2a). We used HyDe^35^ to compute a point estimator of *H* for each individual chestnut tree. The estimated *H* values varied across all *C. henryi* var*. omeiensis* individuals in a range from 11.87 to 44.32% (Fig. 2b), and they also varied across the assembled chromosomes (Fig. 2c). These results together with those of the ADMIXTURE and PCoA analyses provided substantial evidence for genomic mosaicism in this system.

### Design and rationale for testing HHS

The approaches used to test for signals of hybridization by analyzing the ABBA, BABA, and BBAA polymorphic patterns in a four-taxon scenario (e.g., HyDe^35^, Supplementary Fig. 4), which we set as ((s, m), h, outgroup), have been found to be powerful and computationally efficient on a genomic scale^36,37^. These approaches take advantage of phylogenetic control to distinguish incomplete lineage sorting from hybridization between h and m (or s). Here, we used the HyDe^35^ method to identify the barrier genomic regions isolating *C. henryi* var*. henryi* and *C. mollissima*. In our approach, we specify A_sm_ to represent the ancestral lineage of m and s (Supplementary Fig. 4). At a locus (L) arising from hybridization between h and s, gene exchange can occur between h and A_sm_ at the L locus, unless extrinsic barriers (such as geographic barriers) hinder hybridization between them. Following divergence between s and m, some barrier loci, which impede gene exchange between h and m but allow migration between h and s, can evolve from loci that were not previously barriers between h and A_sm_. One major signature of such barrier loci is that gene flow can be found between h and s but not between h and m. When hybridization occurs, mixed barrier alleles sourced from different species can cause maladaptation, due to deleterious effects of mixed variation at one or more barrier loci^38–41^. If hybrid speciation could occur, barrier alleles from one parental population would evolve to be fixed in hybrids (or to be replaced completely by alleles from the other parent in subsequent generations), due to the purging effect of natural selection^6,42^. These two properties thus appear to provide means of identifying barrier genomic regions.

### Mosaic of barrier genomic regions

**Fig. 3:**
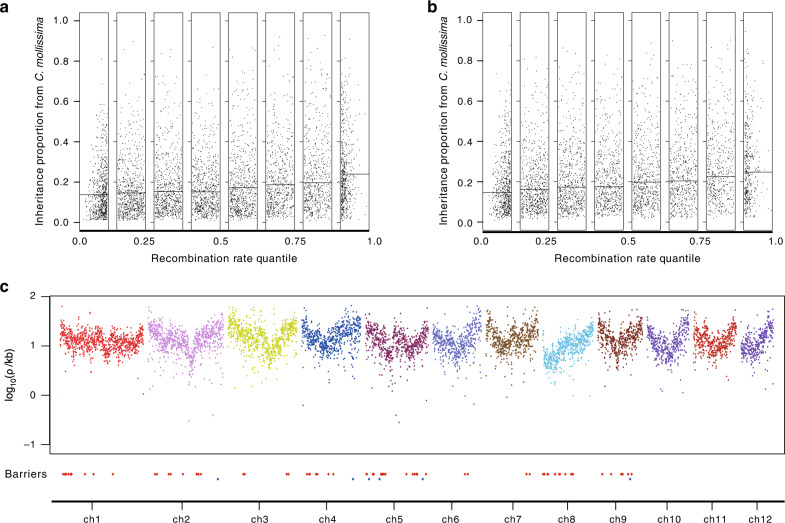
Mosaic of polygenic barriers. **a** Relationship between recombination rates and the proportions of *C. henryi* var. *omeiensis* genetic material inherited from *C. mollissima*. **b** Relationship between recombination rates and the genomic proportions in hybrids (*C. mollissima* × *C. seguinii*) inherited from *C. mollissima*. **c** Manhattan plots of recombination rates across the 12 chromosomes in the *C. mollissima* genome (100-kb nonoverlapping window). To illustrate the distribution of candidate barrier genomic regions, these are listed regions below the Manhattan plots of recombination rates. The red rhombi represent candidate barrier genomic regions derived from *C. henryi* var*. henryi* and the blue rhombi represent regions from *C. mollissima*. Source data are provided as a Source data file.

We examined the correlation between recombination rates (*ρ*) and admixture proportions (using *H*). Several models of divergence under gene flow predicted positive correlations between recombination rates and admixture proportions when multiple barrier loci were involved^43^. We generated a LD-based recombination map for *C. mollissima* using LDhelmet^44^, taking into account specific mutation matrices and the effects of recent changes in population size^45^ (Supplementary Table 10; Supplementary Figs. 5 and 6). The *H* values estimated for genomic regions of *C. henryi* var*. omeiensis* increased with the local recombination rates (Fig. 3a), consistent with the hypothesis that the species barriers between *C. mollissima* and *C. henryi* var*. henryi* are highly polygenic^46^. In addition, we calculated the correlation for the 11 possible hybrids that may result from hybridization between *C. mollissima* and *C. seguinii* (Figs. 1e and 2b), taking these putative hybrids as a single group. The same pattern could also be observed for these hybrids (Fig. 3b), indicating that polygenic barriers might be common in chestnut species.

Using *C. seguinii* as an ingroup, we identified the genomic regions (window size = 100 kbp) with significant introgression between *C. henryi* var*. henryi* and *C. mollissima* (denoted *M*_hm_), using HyDe^35^. Using *C. mollissima* as an ingroup, we identified the genomic regions with significant introgression between *C. henryi* var*. henryi* and *C. seguinii* (denoted *M*_hs_). For loci in *M*_hs_, gene exchange at these loci could occur between *C. henryi* and the ancestor of *C. mollissima* and *C. seguinii*. During the divergence of *C. mollissima* and *C. seguinii*, the barrier genomic regions, which could prevent gene flow between *C. henryi* and *C. mollissima* would fall into *M*_hs_ but not *M*_hm_. Migration between *C. henryi* and the ancestor of *C. mollissima* and *C. seguinii* at the genomic regions in *M*_hs_ but not in *M*_hm_ would have been reduced when *C. mollissima* and *C. seguinii* diverged (Supplementary Fig. 7). Thus genomic regions in *M*_hs_ but not in *M*_hm_ might contain genes preventing gene flow between the two parental lineages.

Local ancestry inference for each genomic region in *M*_hs_ but not in *M*_hm_ suggested that 82 regions in the *C. henryi* var*. omeiensis* genomes were from *C. henryi* var*. henryi* (red rhombus in Fig. 3c), and six from *C. mollissima* (blue rhombus in Fig. 3c). Two additional results further supported this result. First, fixed differences between the two parental lineages could be found in each of 88 candidate barrier regions (*maxF*_ST_ = 1.0). Second, coalescent modeling of 2D-SFS generated from these 88 candidate barrier genome regions suggested that gene flow between parental lineages had reduced after the divergence of *C. mollissima* and *C. seguinii* (Supplementary Table 11). Our results revealed that both *C. mollissima* and *C. henryi* var*. henryi* contributed genomic regions preventing parental introgression to *C. henryi* var*. omeiensis*, therefore directly supporting the HHS hypothesis.

### Genomic distribution of barrier regions

Our analysis of the 82 barrier windows from *C. henryi* var*. henryi* revealed that the number of barrier regions decreased with increasing recombination rate (*F*-statistic = 11.85, *P* < 0.00059), implying that barrier loci may tend to concentrate in genomic regions with low recombination rates. The concentration of barrier loci may render an increase in the density of selection against maladaptive immigrant alleles in regions with low recombination rates and may further lead to the correlation between *H* and recombination rates (Fig. 3a).

### Annotation of barrier regions

In candidate barrier genomic regions, 466 protein coding genes were found and two of them (*bl_011844* on chromosome c2; *bl_014924* on chromosome c8) could be aligned to flowering-time genes in the FLOR-ID database^47^. The *bl_011844* gene was aligned to the *SIZ1* gene, whose product acts as a floral repressor that can repress the SA-dependent pathway and promote *FLC* expression by repressing FLD activity through sumoylation^47,48^. The *bl_014924* gene was aligned to *JMJ14*, whose product can inhibit the floral transition by repressing the floral integrators *FT*, *AP1*, *SOC1* and *LFY* through histone H3K4 demethylation^47,49^.

## Discussion

The present study produced strong genomic evidence for the hypothesis that *C. henryi* var*. omeiensis* originated from hybridization between *C. mollissima* and *C. henryi* var*. henryi*, providing an opportunity to search for potential correlations between candidate barrier genomic regions and traits responsible for RI. The genomic variation identified in *C. henryi* var*. omeiensis* supported its independent evolutionary status (Fig. 1). The simulation-based test and HyDe analysis of population genomic variation showed that *C. mollissima* and *C. henryi* var*. henryi* contributed substantial material to the gene pool of *C. henryi* var*. omeiensis* (Fig. 2), meeting the genomic mosaicism criteria for HHS^10^. Moreover, we identified some candidate barrier genomic regions (Fig. 3), supporting a polygenic barrier model. Barrier effects in candidate regions separating *C. mollissima* and *C. henryi* var*. henryi* would continue to exclude unfit mixed alleles from *C. henryi* var*. omeiensis*. That is, the hybrid lineage has inherited preexisting genetic barriers from its two parents, supporting the HHS hypothesis for *C. henryi* var*. omeiensis*. In particular, our analyses captured the reduction of migration in candidate barrier genomic regions by utilizing phylogenetic control. Our study highlights the importance of using phylogenetic information in identifying molecular segments involved in RI.

When divergence between *C. seguinii* and *C. mollissima* occurred, the genomic landscape of introgression between either species and *C. henryi* would have evolved independently, due to *C. seguinii* and *C. mollissima* beginning to accumulate barrier alleles separately. Depending on the changes of allele or genotype frequencies in hybrids and backcrosses, genome cline analysis and an LD-based genome scan of derived alleles were used to identify candidate barrier loci based on genomic variation in systems without an evolutionary lineage derived from hybridization^8,14^. One extreme scenario is that hybrids fixed genomic regions from one parent, and these might be likely to show the steepest changes in frequency and the strongest LD pattern. It should be noted that frequency changes could also be explained by random drift. To exclude the effects of drift, we employed phylogenetic control and examined the process of reduction in gene flow between parental lineages, with introgression between *C. seguinii* and *C. henryi* var*. henryi* as a background reference. In concordance with morphology^19^, we found that *C. henryi* var*. henryi* contributed more genetic information to the hybrid lineage in candidate barrier genomic regions (82/88). The genes underlying key traits contributing to hybrid speciation were often found to be sourced from different parents in previously reported homoploid hybrid systems^10^, indicating a role for a combinatorial mechanism in initiating species diversification^7–9,42,50^. Consistent with this, our results indicate a role for the assortment of parental barriers in promoting hybrid speciation^6^.

Artificial interbreeding experiments suggested that pre-zygotic barriers may have prevented the incidence of *C. henryi* × *C. mollissima* hybrids^21^. In support of this, our admixture analysis found no obvious hybridization between *C. henryi* var*. henryi* and *C. mollissima* samples (Fig. 1e). The slight difference in flowering periods of *C. henryi* and *C. mollissima*^19^, might indicate partial pre-pollination isolation. Genes inherited from *C. henryi* var*. henryi* may determine the flowering time of the hybrid lineage, because *C. henryi* var*. henryi* contributed more (~75.6%) genetic information to the hybrid taxon. We annotated the candidate barrier genomic regions from *C. henryi* var*. henryi*, and found that two genes were closely related to genes (*SIZ1* and *JMJ14*) controlling the flowering-time trait in *Arabidopsis* plants^47–49^. One focus of future work will be to determine whether there is a direct link between these genes and the difference in flowering time in the two parental taxa. The procedure employed to identify genetic barriers in this study consisted of utilizing the exclusiveness of barrier alleles. Thus, some of the intrinsic genetic incompatibilities, which could further reduce the fitness of hybrids and may have prevented immigrations from parental lineages into the hybrid lineage, may be in the candidate barrier genomic regions. Our results, together with previous studies on chestnut reproduction and phenology^19,21^, suggested that both pre- and post-pollination factors may have contributed to the isolation between *C. henryi* and *C. mollissima*. The relative importance of chromosomal variation, spatial and ecological factors in speciation also still need comprehensive assessment.

The candidate barrier genomic regions are distributed in nine pseudo-chromosomes (Fig. 3c), separated by at least 0.2 Mbp in all cases. This result indicates that the effective migration rates in these candidate regions would be lower than rates in other genomic regions and chromosomes, revealing a heterogeneous landscape of hybrid ancestry across the genome of the hybrid lineage. Recent works have suggested that natural selection together with recombination could influence the genomic distribution of introgressed alleles from the minor parental species^38,51^, by purging the more deleterious effects of mixed genotypes sourced from different species. The positive correlation between admixture proportions (*H*) and recombination rates across the genome in our results supports this prediction (Fig. 3a, b). In contrast to previous reports, our analysis reveals that *C. henryi* var*. omeiensis* has fixed many barrier alleles derived from the major parent *C. henryi* var*. henryi* in genomic regions with low recombination rates. This result suggests that barrier loci from parental lineages would not be uniformly distributed in the genome of the hybrid lineage, tending rather to be densely distributed in genomic regions with less recombination. The correlation between *H* and recombination rates and the landscape of hybrid ancestry across the genome (Fig. 3a) may be shaped by the genomic architecture of reproductive barriers^40,52^. Furthermore, the concentration of barrier regions provides an alternative explanation for the high levels of divergence and differentiation in genomic regions with less recombination.

Due to selection against maladaptive mixed barrier genotypes, alleles present at higher frequencies at barrier loci would be favored by natural selection until fixation occurred. Barrier alleles from the major parent (*C. henryi* var*. henryi*) may be coupled in the regions of hybrid genomes with less recombination, and thus impede the incorporation of barrier alleles from the minor parent (*C. mollissima*). This coupling may also have impeded the maintenance of barriers from the minor parent, with the result that *C. mollissima* contributed far fewer barrier genomic regions (6/88) compared to the genomic composition contributed by *C. mollissima* (*H* = 24.4 ± 1.5%). Furthermore, the six barrier genomic regions sourced from *C. mollissima* should have prevented gene flow from the major parent (*C. henryi* var*. henryi*) to the hybrid lineage, due to the maladaptation of mixed genotypes at barrier loci. Interaction between selection and recombination may therefore have shaped the genomic distribution of parental genetic barriers in the process of forming hybrid species. In future, more studies may be needed in order to assess the relative barrier effect of each candidate locus in this unique hybrid system, and more work in other systems is needed to assess the generality of biased distribution of parental barriers in the genomes of hybrid taxa.

## Methods

### Reference genome assembly

DNA from the Chinese chestnut chosen for the reference genome assembly was extracted and used to construct libraries for Nanopore, Illumina and Hi-C sequencing. We assembled the Nanopore long reads into contigs and used the Illumina short reads to polish the assembled contigs after filtration (see Supplementary note 1). Then we anchored the contigs based on the Hi-C dataset (see Supplementary note 2).

### Gene prediction and functional annotation

We used the ab initio and homology-based methods to perform gene model predictions which were used to generate a set of consensus gene models (see Supplementary Notes 3 and 4). The final gene set was functionally annotated using BLASTP with an *E*-value cutoff of 1e − 5 against the SwissProt and TrEMBL databases. Homologous protein domains were searched for using InterProScan^53^. The GO terms for hits in the above sequence and domain searches were then assigned to the corresponding *C. mollissima* genes. KEGG pathway annotation was conducted using KAAS^54^.

### Population samples and resequencing

We collected young leaves from the four taxa spanning their geographic ranges (Fig. 1 and Supplementary Tables 6–9). We filtered out populations < 5 km distant from villages, cities and man-made chestnut forests, with the aim of minimizing effects due to the domestication of Chinese chestnut trees. We collected fresh leaves from the first year branches, and used silica gel dried leaves for DNA-seq to produce genomic sequences of population samples for each *Castanea* tree. We also collected fresh leaves from eight *Castanopsis* trees and used them as the outgroup in the analyses of population genomics described below. For each chestnut tree, genomic DNA was extracted using a standard protocol. We re-sequenced the individually indexed genomic libraries from all trees to an expected depth of 22× per tree using the Illumina Genome Analyzer (HiSeq 2500), with an insert size of 350 bp and a read length of 150 bp. The raw reads so generated were subject to quality control using FASTX-Toolkit (http://hannonlab.cshl.edu/fastx_toolkit/). Bases with Phred quality score ≤ 20 were defined as low quality. Low-quality bases were masked, and were trimmed if they were at end of the read. Reads with low-quality bases > 95% of read length or with a length of <30 bp were discarded and reads were also trimmed of any adapter and repetitive telomere sequences.

### Mapping and calling

High-quality clean reads were mapped to the *C. mollissima* reference genome using BWA-MEM (0.7.16a-r1181) with default settings^28^. Then we used Picard-tools v1.92 (https://picard.sourceforge.net) to assign read group information containing library, lane, and sample identity, sort the SAM-format files and remove reads marked as duplicates, and generate BAM-format files. We used the HaplotypeCaller tool in Genome Analysis Toolkit (GATK) v4.0.8.1^31^, to call genotypes per site and generated a GVCF format file for each tree. Then we used the GenotypeGVCFs and SelectVariants tools to obtain a list of potential SNPs for each tree. We used a hard-filtering approach to filter raw SNPs for each tree. We determined the filtering rule DP < 8.0 || DP > 60.0 || QD < 2.0 || MQRankSum < −12.5 || ReadPosRankSum < −8.0 || MQ < 40.0 || FS > 60.0 || SOR > 3.0 by calculating the distribution of each statistic and used the VariantFiltration tool to perform the hard filtering. If an SNP site was within a distance < 3 bps of an indel, it was marked as missing. For homozygous-reference calls, we further filtered by minimum and maximum depth (DP < 8.0 and DP > 60.0). Through these filtration steps we generated high-confidence SNPs and also invariant homozygous sites for each tree. The indels identified by the HaplotypeCaller tool, and other sites not satisfying the filtering criteria, were documented as missing. For each tree, the high-confidence SNPs and invariant sites were used to reconstruct FASTA format files.

### Validation of SNP quality

Four trees of *C. mollissima* and three trees of *C. henryi* were used to assess the quality of the SNPs called by the GATK4-pipeline above, based on duplicated re-sequencing. For each of seven trees, two separate DNA samples were sequenced. These datasets were processed in a pipeline identical to that used for processing from quality control to the GATK4-pipeline, ignoring the fact that they were duplicates. Then we compared the genotypes of the two replicate samples for each of the seven trees. We also verified the SNPs using re-sequenced population data from *C. mollissima* and *C. henryi*. The rationale is that if a heterozygotic genotype from one tree genome cannot be detected in the sequencing data from the replicate or be found in population samples, it is treated as an unverified genotype.

### Generating a 2D-SFS dataset

We used ANGSD v0.928^34^ to generate beagle-format files based on filtered reads with a minimal mapping quality score of 30 and filtered bases with a minimal quality score of 20 in BAM-format files. The genomic sequences of eight *Castanopsis* trees, generated by GATK4 and custom python3 scripts, were used as outgroup. We used the -dosaf implementation to calculate the site allele frequency likelihood based on the samtools^55^ genotype likelihood model for all sites, and then employed the realSFS implementation to obtain a maximum likelihood estimate of the unfolded SFS using the Expectation Maximization (EM) algorithm^56^.

### Individual clustering

We performed PCoA analysis using R, based on the variants detected by ANGSD-pipeline (with parameters -minMapQ 30 -minQ 20 -GL 2 -doMajorMinor 1 -doMaf 1 -SNP_pval 2e-6 -doIBS 1 -doCounts 1 -doCov 1 -makeMatrix 1 -minMaf 0.05). We ran ADMIXTURE ver. 1.23^32^ with cross-validation for numbers of genetic clusters (*K*) from 1 to 10 to infer the individual ancestry proportions with default settings. We used PLINK v1.07^57^ to reduce the LD effect. The optimum number of clusters (*K*) was determined using the cross-validation errors. Furthermore, we analyzed our dataset using another robust but time-consuming algorithm implemented in FASTSTRUCTURE^33^.

### Population differentiation

We computed *D*_XY_ and *F*_ST_ for sliding windows (100 kbp nonoverlapping window) using custom python3 scripts, to examine the population differentiation between each pair of four taxa according to the equation proposed by Hudson, Slatkin and Maddison^58–60^. Furthermore, we defined a simple statistic *maxF*_ST_, which is the maximum *F*_ST_/bp in a predefined window (100 kb), to investigate the distribution of fixed differences along the genome. For each taxon, only SNPs with sample size ≥20 were considered for computation.

### The *hhs* test

We defined a statistic *hhs* = *d*_om_/*d*_hm_ in which *d*_om_ was the mean number of nucleotide differences between *C. henryi* var*. omeiensis* and *C. mollissima* and *d*_hm_ was the mean number of nucleotide differences between *C. henryi* var*. henryi* and *C. mollissima*. Given a genomic region with length of *l*, then the equation for *hhs* can be written as

1 $$hhs = \frac{{\mathop {\sum }\nolimits_{k = 1}^l p_{o,k}q_{m,k} + p_{m,k}q_{o,k}}}{{\mathop {\sum }\nolimits_{k = 1}^l p_{h,k}q_{m,k} + p_{m,k}q_{h,k}}}$$

where *p*_*x,k*_ and *q*_*x,k*_ denote the frequencies of two alleles at site *k* in taxon *x*. If the genetic compositions of *C. henryi* var*. omeiensis* were fully derived from *C. henryi* var*. henryi*, *hhs* would be equal to 1.0; if *C. mollissima* contributed any genetic information to *C. henryi* var*. omeiensis*, *hhs* would be <1.0 at some loci. We used the bootstrap method with 1000 replications to test whether *hhs* = 1.0, applying the sliding-window approach with a window size of 100 kbp. For each replicate, we sampled windows randomly using the R function sample() and computed the *hhs* value for each window. The *hhs* value for each replicate was computed as the mean of these sampled windows. We then summarized *hhs* values of all replicates.

### Coalescent test

We employed fastsimcoal2^61,62^, to estimate the *H* parameter in a coalescent model based on the 2D-SFS format datasets. The *H* parameter^63^ here represents the relative contribution from the *C. mollissima* genomes (Figs. 2 and S3.1.1). We removed the *C. seguinii* samples when testing for genomic mosaicism. We compared three models using the Akaike Information Criterion (AIC) method (Supplementary Fig. 3). In model 1 (*H* = 0), *C. henryi* var*. omeiensis* diverged from *C. henryi* var*. henryi*. In model 2 (*H* = 1), *C. henryi* var*. omeiensis* diverged from *C. mollissima*. Model 3 (0 < *H* < 1) represents a HHS model in which the genome of *C. henryi* var*. omeiensis* was derived from hybridization between *C. henryi* var*. henryi* and *C. mollissima*. We calculated likelihoods for each model based on 300,000 coalescent simulations. The likelihood and parameters were estimated in 50 ECM cycles. The rate was set to 5 × 10^−9^ substitutions per site per year^64^, and 15 years per generation was assumed. The -C parameter, that is the minimum size of entry of the observed and simulated SFS, was set to 5. Standard deviations were determined from estimates from 20 duplicated computations with different seed numbers.

### HyDe analyses

The HyDe^35^ program detects signal of hybridization between two non-sister lineages, using the unbiased statistic of the *H* parameter proposed by Kubatko and Chifman^37^. We combined the HyDe^35^ program with a sliding-window approach to estimate the *H* parameter for each nonoverlapping window with sizes of 100 kbp and 1 Mbp across the genome. We used run_hyde.py to examine whether genomic signal of hybridization could be detected in samples of *C. henryi* var. *omeiensis* based on the sliding-window approach (window sizes: 100 kbp and 1 Mbp). Then we used individual_hyde.py to estimate the *H* parameter at an individual scale for each window (1 Mbp) and summarized all results to compute the *H* parameter for each *C. henryi* var. *omeiensis* individual. For each of the 11 hybrids possibly produced by *C. seguinii* × *C. mollissima*, we also performed the HyDe analysis with *C. seguinii* and *C. mollissima* as parents.

### PSMC and SMC++ analyses

We applied the Pairwise Sequentially Markovian Coalescent (PSMC) model^65^ to reconstruct demographic histories for *C. mollissima* and its relatives. We ran PSMC on each individual tree of the four taxa collected for this study based on the high-confidence sequences generated by our GATK4-pipeline. We set the ratio of *θ* to *ρ* (-r 2); the -g parameter (number of years per generation) to 15; the -p parameter to 64 × 2; and the rate to 5 × 10^−9^ substitutions per site per year^64^. We presented all results for each individual of the four lineages using one coordinate system to ensure that the results were reliable, using the program psmc_plot.pl from the PSMC package. To resolve the recent demographic histories clearly and robustly, we also inferred the demographic history using SMC++ (https://github.com/popgenmethods/smcpp). For each individual sample, we generated a whole-genome diploid consensus sequence with the high-confidence SNPs and invariant homozygous sites with the GATK4-pipeline. To test the effects of the -d parameter, we ran SMC++ for each tree and the corresponding taxon. That is, each tree was set as a discrete sample and resequencing samples from each taxon were used in each SMC++-estimate analysis. We set the -spline parameter to the piecewise type; the -knots parameter to 40 and the -timepoints parameter from 1 to 5,000,000. Finally, we used SMC++-plot to summarize and depict the demographic histories of the four taxa.

### Recombination map construction

We used LDhelmet v1.9^44^ to generate a fine-scale recombination map for *C. mollissima*, based on the genomic sequences generated by the GATK4-pipeline. To infer recombination rates, the LDhelmet program requires: (1) the population mutation rate (*θ*), (2) phased haplotypes, (3) the block penalty, (4) the ancestral allelic state per SNP used as prior, and (5) a mutation matrix for the focused species. Recent studies have shown that past bottleneck events may affect the estimation of recombination rates^45^, thus we took recent demographic changes in *C. mollissima* into account using LDpop^45^. LDhelmet and LDpop can only process up to 50 haplotypes, so we retained 25 trees of *C. mollissima* with long genome sequences (lower proportion of missing bases throughout the genome). We used the observed heterozygosity in 43 *C. mollissima* trees as the population mutation rate (here we used 0.0066). We used beagle v5^66,67^ to generate the phased haplotypes for variants in each of the assembled contigs. To reduce the effects of missing bases, we retained only those SNPs with no more than 20% missing bases. In two recent studies^38,68^, simulation-based analyses showed that LDhelmet performed better in simulations with a block penalty of 5 than with a higher block penalty, such as 50. We used a block penalty of 5 in our analysis. We applied a parsimony-based method^44^ to infer the ancestral allele distribution at each site in *C. mollissima* by comparison with the aligned outgroup reference genomes of *C. henryi* var. *henryi*, var. *omeiensis* and *C. seguinii*. For each dimorphic site in *C. mollissima* genomes, if the alleles of the three outgroups were not all missing for this site and if they collectively exhibited precisely one of the four possible nucleotides, and if this allele agreed with one of the two observed in *C. mollissima*, then this was designated as the ancestral allelic state. Otherwise, it was considered to be unknown and we used the uniform distribution as a prior over the ancestral allele. To consider uncertainty in ancestral allele reconstruction, we set the prior for the putative ancestral allele as 0.91 and the priors for the other three bases as 0.03. When estimating the mutation matrix for *C. mollissima*, we considered only biallelic SNPs. We then followed the Chan et al.^44^ approach to estimate the mutation matrix for this species (Supplementary Table 10). We used LDpop to generate the lookup table, which was used in LDhelmet. We computed a likelihood lookup table for a grid of *ρ* values (-rh 0.0, 0.01, 1.0, 1.0, 100.0), for each set of haplotypes (-*n* 50), for each of 422 contigs. We set the -th parameters to 0.0066 and set the --approx option. The SMC++ results for *C. mollissima* were set in LDhelmet. Then we converted the format of the lookup table using convert_table from the LDhelmet package. We used the rjMCMC procedure with a burn-in of 100,000 and ran the Markov chain for 1 million iterations. After excluding SNPs for which recombination rate was estimated to be implausibly higher than 20 × *θ* (here we used 0.132), similar to the Schumer et al.^38^ settings, we finally summarized recombination rates in 100 kb windows (Fig. 3).

### Correlation between *ρ* and *H*

We examined the possible correlation between the recombination rate and genomic contribution from *C. mollissima* to *C. henryi* var*. omeiensis* (*H*) using the R function lm(). We also calculated the correlation between the recombination rate and genomic contribution from *C. mollissima* to *C. seguinii*.

### Barrier genomic regions

We used HyDe^35^ and sliding-window analysis (100 kbp) to identify the windows that showed significant hybridization between *C. seguinii* and *C. henryi* var*. henryi*, with *C. mollissima* as the ingroup (*P* < 0.05). Then, with *C. seguinii* as the ingroup, we identified those loci that showed significant hybridization between *C. mollissima* and *C. henryi* var*. henryi*. For loci in *M*_hs_ and *M*_hm_, gene exchange between *C. henryi* and A_sm_ could occur if migration rates were positive. We used the HyDe program and sliding-window analysis with a window size of 100 kbp to perform local ancestry analysis; that is, to examine the source of genetic composition for each *C. henryi* var*. omeiensis* genomic window. For the test with the tree topology ((henryi, (omeiensis, mollissima)), outgroup) in a genomic window, if there was no significant signal of gene flow, this genomic window for *C. henryi* var*. omeiensis* was determined to be derived from *C. mollissima*. For the test with the tree topology ((mollissima, (omeiensis, henryi)), outgroup) in a genomic window, if there was no significant signal of gene flow, this genomic window for *C. henryi* var*. omeiensis* was determined to be sourced from *C. henryi* var*. henryi*.

### Testing barrier regions

We used the coalescence-based method implemented in fastsimcoal2^61^ to test whether the rate of migration between *C. mollissima* and *C. henryi* var*. henryi* (*M*_hm_) was lower than the ancestral migration rate (*M*_anc_) before time *t*_*2*_ in the candidate barrier genomic windows (Supplementary Fig. 7). Two models were designed. In model A, *M*_hm_ < *M*_anc_; and in model B, *M*_hm_ ≥ *M*_anc_ or *M*_hm_ = *M*_anc_. We used the ANGSD-pipeline to generate the unfolded 2D-SFS format datasets for *C. mollissima* and *C. henryi* var*. henryi* in candidate barrier genomic windows. In this coalescent analysis using fastsimcoal2, we calculated the likelihood of each model based on 300,000 coalescent simulations. The likelihood and parameters were estimated in 50 ECM cycles. The -C parameter was set to 5 and the AIC values were used to compare the two models. For robustness, we performed 20 duplicated tests with different seed numbers for the dataset generated from the 88 candidate barrier genomic windows.

### Relationship between barriers and *ρ*

We analyzed the relationship between *ρ* and the number of barrier windows sourced from *C. henryi* var*. henryi* within the genome of hybrid lineage using the R function lm(). The barrier windows from the major parent were set to be 1 and the other windows to be 0.

### Annotation of barrier regions

We annotated the functions of genes in the candidate barrier genomic regions, using the FLOR-ID database^47^. The genes that passed an *E*-value filtration (1e − 10) were further manually checked through comparison of their top blast hits in the FLOR-ID and SwissProt databases.

### Reporting summary

Further information on research design is available in the Nature Research Reporting Summary linked to this article.

## Supplementary information

Supplementary Information

Reporting Summary

## Data Availability

Data supporting the findings of this work are available within the paper and its Supplementary Information files. A reporting summary for this article is available as a Supplementary Information file. The datasets generated and analyzed during the current study are available from the corresponding author upon request. All raw sequence reads have been deposited in the NCBI SRA database under BioProject accession number PRJNA540917 [https://www.ncbi.nlm.nih.gov/bioproject/540917]. Assembly and annotation of *Castanea mollissima* genome are available at GitHub [https://github.com/yongshuai-sun/hhs-omei]. The source data underlying Figs. 1b–g, 2b,c, and 3, as well as Supplementary Figs. 2 and 5–7 are provided as a Source data file. Source data file is also available at GitHub [https://github.com/yongshuai-sun/hhs-omei].

## Source data

Source Data

## Code availability

The scripts used in this study are available at GitHub [https://github.com/yongshuai-sun/hhs-omei].
