# Supplementary material for: Genomic basis of homoploid hybrid speciation within chestnut trees
Source: Nat Commun. 2020 Jul 6;11:3375. doi: 10.1038/s41467-020-17111-w (PMC7338469; doi:10.1038/s41467-020-17111-w)
Supplement: Supplementary file 1 — Supplementary Information [file 41467_2020_17111_MOESM1_ESM.pdf]

Genomic basis of homoploid hybrid speciation within chestnut trees

Sun *et al.*

Supplementary Note 1. Nanopore sequencing and assembly

Fresh leaves of chestnut trees were collected and used to extract high-quality genomic DNA by the CTAB method[1]. For Nanopore long read sequencing of the *C. mollissima* sample, the large (>20 Kb) DNAs were used directly for library construction, using a Ligation Sequencing Kit 1D (SQK-LSK108) from Oxford Nanopore and then the DNAs were sequenced on a GridION X5 sequencer. Sequencing adapters and low-quality nucleotides (if mean quality score <7) were removed.

We used two methods to assemble the Nanopore long reads (SMARTdenovo, https://github.com/ruanjue/smartdenovo; WTDBG, https://github.com/ruanjue/wtdbg). The genome sequence assembled by WTDBG was too large (870 Mb), so we discarded it. The consensus sequences produced by SMARTdenovo were further corrected by NextDenovo (https://github.com/Nextomics/NextDenovo) and polished by NextPolish (https://github.com/Nextomics/NextPolish) based on the Illumina short reads of the *C. mollissima* sample.

For Illumina short reads sequencing of the *C. mollissima* sample, we constructed paired-end libraries with an insert size of ~350 bp according to the manufacturer's standard protocol (Illumina) and carried out sequencing on the Illumina HiSeq 2500 platform to produce short reads. The raw Illumina paired-end reads were processed to remove duplications, adaptors, and low-quality bases using Super-Deduper[2] and Trimmomatic V0.35 [3], and the mate-pair reads were cleaned using NextClip V1.3.1 with default parameters[4]. Based on the short reads, we used k-mer frequency analysis to estimate the genome size with Jellyfish[5], with a k-mer length setting of 17.

Supplementary Note 2. Hi-C scaffolding

For high-throughput chromosome conformation capture (Hi-C) sequencing of the *C. mollissima* sample, fresh chestnut leaves were picked and fixed in formaldehyde (1% volume/volume [v/v]) for 10 min at room temperature. Chromatin was cross-linked then and digested using the restriction enzyme HindIII. The 5' overhangs were filled with biotinylated nucleotides, and free blunt ends were then ligated. After ligation,

cross-linking was reversed and the DNA of the *C. mollissima* sample was purified away from protein. Purified DNA of the *C. mollissima* sample was treated to remove biotin that was not internal to ligated fragments. Then, the DNA of the *C. mollissima* sample was sheared into fragment sizes of ~350 bp and sequenced using the Illumina HiSeq 2500 platform. After filtration of adapters, we removed reads of low quality (defined as: more than 5 missing bases; 17% or more bases with Phred quality score ≤ 15). We used bowtie V2.3.2[6] to align reads to the assembled contigs above. We used HiC-Pro[7] to evaluate the quality of Hi-C data and filter the cleaned Hi-C reads of the *C. mollissima* sample. The contigs were anchored to chromosomes using LACHESIS[8], with parameters CLUSTER_MIN_RE_SITES=100; CLUSTER_MAX_LINK_DENSITY=2.5; CLUSTER_NONINFORMATIVE_RATIO=1.4; ORDER_MIN_N_RES_IN_TRUNK=60; ORDER_MIN_N_RES_IN_SHREDS=60.

Supplementary Note 3. Identification of repetitive elements

We identified repetitive elements using a *de-novo* identification method employed in RepeatModeler v2.0 (www.repeatmasker.org), and the homolog-based approach in RepeatMasker v4.0 to identify repetitive elements homologous to those in the Dfam, Repbase and RepeatPeps libraries[9-11]. Then, we used LTR_Finder, LTR_harvest and other tools in LTR_retriever software to reannotate LTR retroelements[12-14]. Overlapping repetitive elements were merged with bedtools v2.28.0[15].

Supplementary Note 4. Gene prediction

Putative protein-coding genes were predicted following the Funannotate pipeline (https://funannotate.readthedocs.io). Briefly, *ab-initio* gene model predictions were carried out by Augustus v3.2[16,17], Snap (http://korflab.ucdavis.edu/), GlimmerHMM v3[18] and GeneMark-ET[19]; homology-based gene model predictions were performed by GeMoMa[20] based on the published *Castanea mollissima* genome annotation. RNAseq reads (NCBI SRA accession SRR8731962) were filtered with Trimmomatic v0.35[3] and assembled with Trinity v2.8.5[21]. We used PASA (http://pasa.sourceforge.net), GenomeThreader[22] and Hisat2[23] to align transcripts, Uniprot-sprot protein sequences and RNAseq reads respectively. The above

predictions were then passed to EVM v1.1 to generate a set of consensus gene models[24].

Supplementary Table 1. Comparison of assembly quality for two genome assemblies of *Castanea mollissima*.

Genome assembly statistics	Value (in this study)	Value Xing *et al.*[25]
Total length	773,991,346 bp	785,529,252 bp
No. of contigs	422	2,707
Longest contig length	41,151,384 bp	6,584,328 bp
N50 length (contigs)	**5,875,430** bp	944,461 bp
N90 length (contigs)	889,749 bp	133,678 bp
Counts of N50 (contigs)	29	235
Counts of N90 (contigs)	163	1,024

Supplementary Table 2. Summary of repetitive elements identified by RepeatMasker in the *C. mollissima* genome.

	Number of sequences	Length	Percentage of sequences
Retroelements	126104	78123871	10.09
SINEs:	793	97257	0.01
Penelope	0	0	0.00
LINEs:	27140	11538377	1.49
CRE/SLACS	23	1085	0.00
L2/CR1/Rex	0	0	0.00
R1/LOA/Jockey	0	0	0.00
R2/R4/NeSL	0	0	0.00
RTE/Bov-B	3086	778190	0.10
L1/CIN4	24031	10759102	1.39
LTR elements:	98171	66488237	8.59
BEL/Pao	0	0	0.00
Ty1/Copia	36628	24585221	3.18
Gypsy/DIRS1	56600	39221389	5.07
Retroviral	0	0	0
DNA transposons	102750	11682993	1.51
hobo-Activator	17831	4378300	0.57
Tc1-IS630-Pogo	1940	78419	0.01
En-Spm	0	0	0.00
MuDR-IS905	0	0	0.00
PiggyBac	0	0	0.00
Tourist/Harbinger	8485	1154851	0.15
Rolling-circles	13962	2554741	0.33
Unclassified	755	191250	0.02
Total interspersed repeats:		89998114	11.63
Small RNA:	728	91460	0.01
Satellites:	517	50062	0.01
Simple repeats:	0	0	0.00
Low complexity:	0	0	0.00

Supplementary Table 3. Summary of repetitive elements identified by RepeatModeler in the *C. mollissima* genome.

	Number of sequences	Length	Percentage of sequences
Retroelements	155070	172096204	22.23
SINEs:	0	0	0.00
Penelope	0	0	0.00
LINEs:	52534	29598556	3.82
CRE/SLACS	0	0	0.00
L2/CR1/Rex	0	0	0.00
R1/LOA/Jockey	0	0	0.00
R2/R4/NeSL	0	0	0.18
RTE/Bov-B	7195	1400379	0.10
L1/CIN4	44283	27932016	3.61
LTR elements:	102536	142497648	18.41
BEL/Pao	121	14442	0.00
Ty1/Copia	53070	55405176	7.16
Gypsy/DIRS1	40064	79401503	10.26
Retroviral	0	0	0
DNA transposons	34503	16688771	2.16
hobo-Activator	16329	5214386	0.67
Tc1-IS630-Pogo	0	0	0.00
En-Spm	0	0	0.00
MuDR-IS905	0	0	0.00
PiggyBac	0	0	0.00
Tourist/Harbinger	4342	1896034	0.24
Rolling-circles	9661	4902734	0.63
Unclassified	939284	242624970	31.35
Total interspersed repeats:		431409945	55.74
Small RNA:	1645	553070	0.07
Satellites:	0	0	0.00
Simple repeats:	315464	11138494	1.44
Low complexity:	47454	2268688	0.29

Supplementary Table 4. Gene prediction statistics for the *C. mollissima* genome.

Software	Weight	Number of gene models
August	1	34727
Augustus HiQ	2	1775
GeneMark	1	115794
GlimmerHMM	1	173919
SNAP	1	209348
PASA	6	47046
GeMoMa	1	98425

Supplementary Table 5. Summary of the functional annotation of 45011 protein coding genes in the *C. mollissima* genome.

Annotation database	Annotated number	Percentage (%)
SwissProt	25452	56.55
TrEMBL	35332	78.50
InterPro	37544	83.41
GO	35630	79.16
KEGG Pathway	9359	20.79
Total	38928	86.49

Supplementary Table 6. Sampling information for each *C. mollissima* tree.

Sample name	Latitude	Longitude	No. of high-confidence sites	No. of heterozygous sites	No. of missing bases	Observed heterozygosity
m7_1	N31°11'59"	E116°0'38"	570742007	3707657	203249339	0.65%
m7_2	N27°17'49"	E104°47'8.3"	522308199	2606089	251683147	0.50%
m7_3	N27°45'26"	E106°49'15"	562017772	3778988	211973574	0.67%
m7_4	N26°59'11"	E106°28'42"	532302924	3501721	241688422	0.66%
m7_5	N31°44'2"	E109°38'37"	552102008	3809841	221889338	0.69%
m7_6	N31°43'44"	E109°45'12"	545413068	3818000	228578278	0.70%
m7_7	N31°59'2"	E110°13'12"	552132054	3957800	221859292	0.72%
m7_8	N31°52'35"	E110°36'5"	541691204	3990168	232300142	0.74%
m7_9	N30°44'12"	E110°18'52"	541917546	3834239	232073800	0.71%
m7_10	N27°38'44"	E109°49'46"	550499295	3707115	223492051	0.67%
m7_11	N29°25'40"	E110°58'58"	560352866	3869141	213638480	0.69%
m7_12	N29°3'11"	E113°32'14"	591563995	3722198	182427351	0.63%
m7_13	N26°8'4"	E113°48'32"	554861054	3694940	219130292	0.67%
m7_14	N25°30'21"	E113°54'51"	529580541	3297121	244410805	0.62%
m7_15	N27°24'51"	E109°58'55"	548403007	3573249	225588339	0.65%
m7_16	N31°18'7"	E119°40'15"	547400705	3685792	226590641	0.67%
m7_17	N28°16'41"	E113°37'43"	557025720	3396141	216965626	0.61%
m7_18	N28°38'60"	E114°54'7"	552877733	3743615	221113613	0.68%
m7_19	N29°14'10"	E117°54'32"	592970264	3895654	181021082	0.66%
m7_20	N26°10'25"	E114°33'22"	566265530	3720066	207725816	0.66%
m7_21	N23°11'54"	E104°52'12"	539418831	3359302	234572515	0.62%
m7_22	30°26'4"	E119°35'13"	545270958	3547029	228720388	0.65%
m7_23	N29°0'20"	E120°31'9"	588299158	3647102	185692188	0.62%
m7_24	N27°52'04"	E119°49'40"	541353389	3624997	232637957	0.67%
m7_25	N31°57'39"	E108°35'13"	555839860	3655137	218151486	0.66%
m7_26	N31°48'39"	E109°04'26"	545212445	3728296	228778901	0.68%
m7_27	N33°54'49"	E105°59'40"	565916720	4067957	208074626	0.72%
m7_28	N33°54'49"	E105°59'40"	549536838	3690186	224454508	0.67%
m7_29	N27°53'10"	E108°47'48"	584870016	3396872	189121330	0.58%
m7_30	N27°53'10"	E108°47'48"	560264444	3793601	213726902	0.68%
m7_31	N26°13'59"	E107°55'08"	553333070	3769171	220658276	0.68%
m7_32	N26°13'59"	E107°55'08"	519097967	3384447	254893379	0.65%
m7_33	N26°26'36"	E111°0'26"	559469681	3690586	214521665	0.66%
m7_34	N26°26'36"	E111°0'26"	558588069	3933412	215403277	0.70%
m7_35	N33°22'21"	E106°12'21"	517962008	3327264	256029338	0.64%
m7_36	N33°22'21"	E106°12'21"	547324730	3795303	226666616	0.69%
m7_37	N29°35'16"	E103°17'18"	545449456	3707953	228541890	0.68%
m7_38	N29°35'16"	E103°17'18"	545809790	3196931	228181556	0.59%
m7_39	N31°16'18"	E115°59'38"	537191914	3909693	236799432	0.73%
m7_40	N31°16'18"	E115°59'38"	557332786	3880544	216658560	0.70%
m7_41	N27°17'	E104°47'	570601025	3732892	203390321	0.65%
m7_42	N27°45'	E106°49'	547772612	3739079	226218734	0.68%
m7_43	N32°52'51"	E107°49'60"	571101787	3858436	202889559	0.68%

Supplementary Table 7. Sampling information for each *C. henryi* var. *henryi* tree.

Sample name	Latitude	Longitude	No. of high-confidence sites	No. of heterozygous sites	No. of missing bases	Observed heterozygosity
h7_01	N26°39'43"	E117°33'49"	461373964	3672669	312617382	0.80%
h7_02	N26°39'43"	E117°33'49"	449710156	3447569	324281190	0.77%
h7_03	N26°39'43"	E117°33'49"	471016087	3550445	302975259	0.75%
h7_04	N28°1'26"	E108°45'52"	468509572	4401177	305481774	0.94%
h7_05	N28°1'26"	E108°45'52"	440516104	3862828	333475242	0.88%
h7_06	N26°19'31"	E108°1'18"	450599087	4019797	323392259	0.89%
h7_07	N26°19'31"	E108°1'18"	445825050	3780122	328166296	0.85%
h7_08	N26°19'31"	E108°1'18"	459317726	4262963	314673620	0.93%
h7_09	N31°52'35"	E110°36'5"	464782833	3876914	309208513	0.83%
h7_10	N31°52'35"	E110°36'5"	451847271	4299011	322144075	0.95%
h7_11	N30°43'54"	E110°18'16"	444465736	4015897	329525610	0.90%
h7_12	N30°43'54"	E110°18'16"	438690134	3412361	335301212	0.78%
h7_13	N29°0'38"	E109°55'06"	490293641	4315259	283697705	0.88%
h7_14	N29°25'40"	E110°58'58"	448518559	4297723	325472787	0.96%
h7_15	N29°3'11"	E113°32'14"	489371990	4147459	284619356	0.85%
h7_16	N28°16'41"	E113°37'43"	448871955	4150474	325119391	0.92%
h7_17	N26°8'4"	E113°48'32"	460558849	3668762	313432497	0.80%
h7_18	N26°26'36"	E111°0'26"	459906471	3838423	314084875	0.83%
h7_19	N27°24'51"	E109°58'55"	454162952	4293360	319828394	0.95%
h7_20	N28°40'2"	E114°44'7"	490685985	3886581	283305361	0.79%
h7_21	N30°26'4"	E119°35'13"	443667662	3590636	330323684	0.81%
h7_22	N29°0'20"	E120°31'9"	445924953	3872337	328066393	0.87%
h7_23	N27°52'30"	E119°08'25.6"	462631380	2712473	311359966	0.59%
h7_24	N31°48'39"	E109°04'26"	446025357	3744315	327965989	0.84%

Supplementary Table 8. Sampling information for each *C. henryi* var. *omeiensis* tree.

Sample name	Latitude	Longitude	No. of high-confidence sites	No. of heterozygous sites	No. of missing bases	Observed heterozygosity
o7_1	N29°35'16"	E103°17'18"	479040224	6628868	294951122	1.38%
o7_2	N29°35'22"	E103°22'56"	464410264	4144702	309581082	0.89%
o7_3	N29°35'23"	E103°22'57"	472241836	4530186	301749510	0.96%
o7_4	N29°35'24"	E103°22'58"	460641015	4085421	313350331	0.89%
o8_01	N29°34'44"	E103°23'11"	477735054	4804066	296256292	1.01%
o8_02	N29°34'44"	E103°23'11"	450372499	4016571	323618847	0.89%
o8_03	N29°34'44"	E103°23'11"	472864333	4391174	301127013	0.93%
o8_04	N29°34'44"	E103°23'11"	464350640	4240950	309640706	0.91%
o8_05	N29°34'56"	E103°23'06"	508629818	7909021	265361528	1.55%
o8_06	N29°34'56"	E103°23'06"	465639951	4377841	308351395	0.94%
o8_07	N29°34'56"	E103°23'06"	456802111	3922734	317189235	0.86%
o8_08	N29°34'56"	E103°23'06"	462240844	4373510	311750502	0.95%
o8_09	N29°34'56"	E103°23'06"	462680989	4123521	311310357	0.89%
o8_10	N29°35'12"	E103°22'59"	453760082	3945910	320231264	0.87%
o8_11	N29°35'12"	E103°22'59"	397162243	3816785	376829103	0.96%
o8_12	N29°35'12"	E103°22'59"	469303626	5866881	304687720	1.25%
o8_13	N29°35'22"	E103°22'56"	439380481	3966063	334610865	0.90%
o8_14	N29°35'22"	E103°22'56"	515221031	8722588	258770315	1.69%
o8_15	N29°35'22"	E103°22'56"	460882909	3725157	313108437	0.81%
o8_16	N29°35'16"	E103°17'18"	510769536	7041891	263221810	1.38%

Supplementary Table 9. Sampling information for each *C. seguinii* tree.

Sample name	Latitude	Longitude	No. of high-confidence sites	No. of heterozygous sites	No. of missing bases	Observed heterozygosity
s7_01	N27°28'50"	E104°51'2"	491002223	3567482	282989123	0.73%
s7_02	N27°28'50"	E104°51'2"	492370576	3686093	281620770	0.75%
s7_03	N30°54'5"	E116°16'14"	474634420	3983384	299356926	0.84%
s7_04	N31°11'59"	E116°0'38"	489102692	4106887	284888654	0.84%
s7_05	N31°16'18"	E115°59'38"	474160345	4067299	299831001	0.86%
s7_06	N29°28'20"	E118°9'4"	490313162	3960697	283678184	0.81%
s7_07	N26°40'7.7"	E104°46'5.6"	491543074	3887879	282448272	0.79%
s7_08	N27°45'26"	E106°49'15"	490950378	4366180	283040968	0.89%
s7_09	N27°45'26"	E106°49'15"	509620360	5473503	264370986	1.07%
s7_10	N25°52'43"	E106°13'32"	501537000	4281769	272454346	0.85%
s7_11	N26°13'59"	E107°55'08"	490250460	5950090	283740886	1.21%
s7_12	N26°19'31"	E108°1'18"	473258703	3897652	300732643	0.82%
s7_13	N31°49'3"	E114°4'42"	492477340	4147051	281514006	0.84%
s7_14	N31°49'3"	E114°4'42"	481313562	4011638	292677784	0.83%
s7_15	N29°3'54"	E113°35'26"	494875509	3693239	279115837	0.75%
s7_16	N25°31'51"	E113°27'30"	480053586	4005889	293937760	0.83%
s7_17	N26°30'17"	E110°56'29"	512595931	5818045	261395415	1.14%
s7_18	N26°30'17"	E110°56'29"	498034642	4709612	275956704	0.95%
s7_19	N31°16'58"	E119°46'55"	496668952	4716012	277322394	0.95%
s7_20	N28°16'41"	E113°37'43"	500777481	4065136	273213865	0.81%
s7_21	N28°40'2"	E114°44'7"	490733281	4318669	283258065	0.88%
s7_22	N28°38'60"	E115°7'53"	478310769	3850703	295680577	0.81%
s7_23	N26°30'27"	E116°15'37"	510335136	4511877	263656210	0.88%
s7_24	N26°10'25"	E114°33'22"	494328988	4091157	279662358	0.83%
s7_25	N30°26'4"	E119°35'13"	496135014	4133930	277856332	0.83%
s7_26	N29°0'20"	E120°31'9"	504595071	4485771	269396275	0.89%
s7_27	N29°0'20"	E120°31'9"	498362011	5005579	275629335	1.00%
s7_28	N30°44'12"	E110°18'52"	500004423	4316463	273986923	0.86%

Supplementary Table 10. Mutation matrices for *C. mollissima* following the method of Chan *et al.*[26].

	**A**	**C**	**G**	**T**
A	0.673148	0.053624	0.17474	0.098488
C	0.18217	0.00065	0.07571	0.741469
G	0.741269	0.076225	0	0.182506
T	0.099325	0.175132	0.053945	0.671598

Supplementary Table 11. AIC scores of model A and B.

Duplicate code	AIC	
	Model A ($M_{hm} < M_{anc}$)	Model B ($M_{hm} >= M_{anc}$)
duplicate 1	13541420	21421351
duplicate 2	13545533	21870169
duplicate 3	13547745	18625330
duplicate 4	13555198	18320133
duplicate 5	13555547	16283226
duplicate 6	13559190	17000360
duplicate 7	13563053	21452227
duplicate 8	13563053	21452227
duplicate 9	13565076	20675993
duplicate 10	13565099	16215901
duplicate 11	13565099	16215901
duplicate 12	13568960	21574060
duplicate 13	13568960	21574060
duplicate 14	13569722	15891021
duplicate 15	13569722	15891021
duplicate 16	13570588	21723945
duplicate 17	13576897	21738839
duplicate 18	13576897	21738839
duplicate 19	13577305	15929488
duplicate 20	13579794	16164942

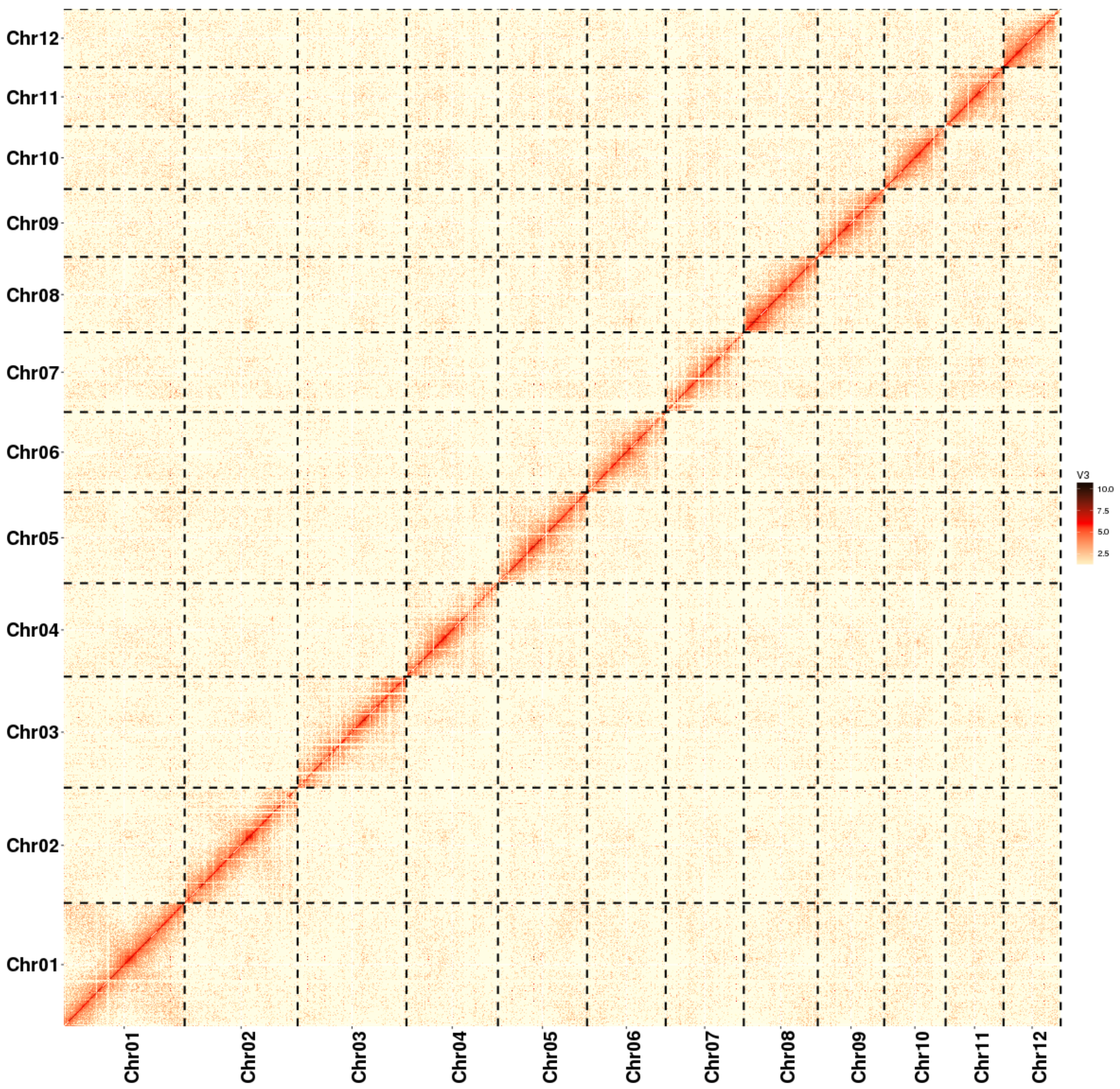

Supplementary Figure 1. Chromatin interaction map for *C. mollissima* based on its Hi-C library. Each group (ChrX, X=1, 2, 3, … , 12) represents a chromosome-scale scaffold.

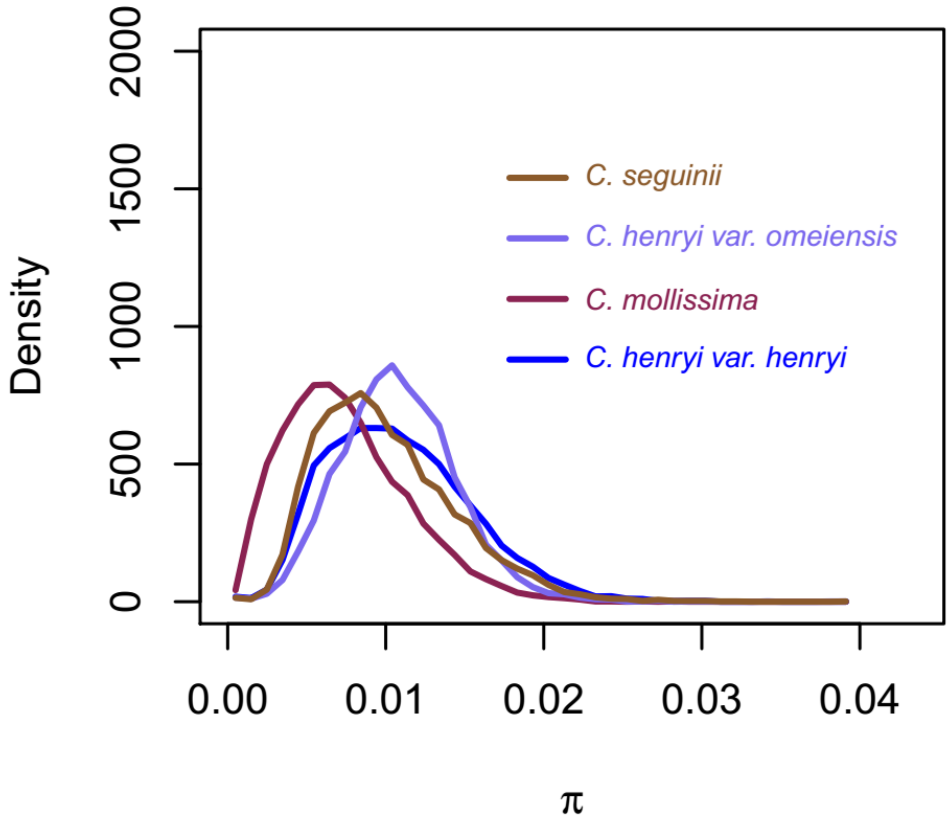

Supplementary Figure 2. Frequency polygon of *π* per base-pair with a sliding window approach. Window size = 100 Kbp. Source data are provided as a Source Data file.

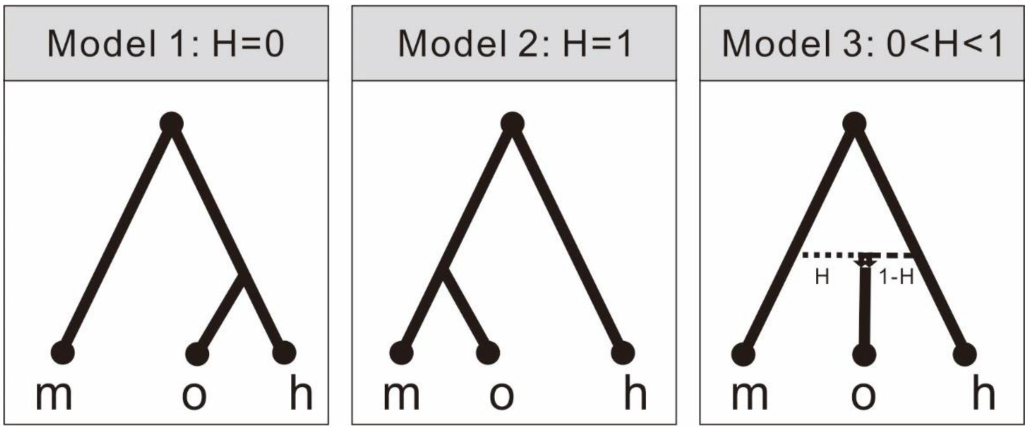

Supplementary Figure 3. Three models representing three hypotheses for the origin of *C. henryi* var. *omeiensis* (o). Model 3 represents the HHS hypothesis, in which the H parameter indicates the genomic contribution from *C. mollissima* (m) and the remainder (1-H) represents the genomic contribution from *C. henryi* var. *henryi* (h).

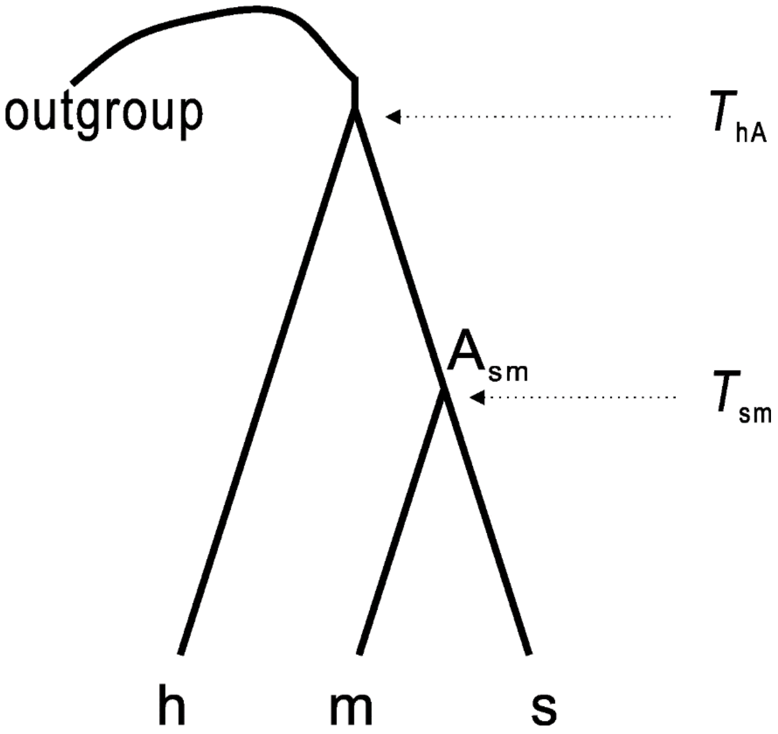

Supplementary Figure 4. The evolutionary model used to capture the reduction of migration between h and m by utilizing the s lineage. A_{sm} represents the ancestor of s and m. The divergence between s and m occurred at T_{sm} and the divergence between h and A_{sm} occurred at T_{hA}. *C. mollissima* = m, *C. seguinii* = s, *C. henryi* var. *henryi* = h.

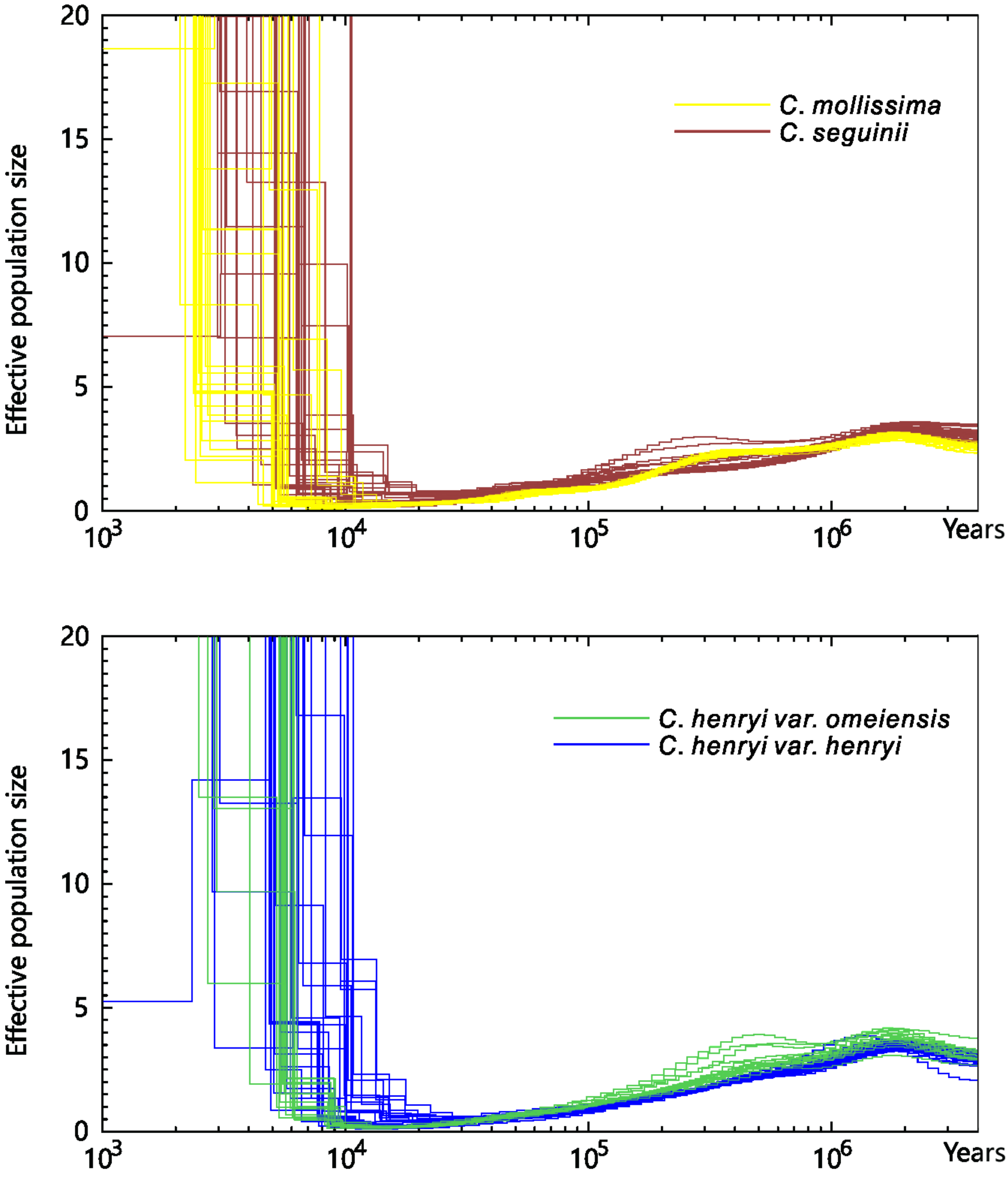

Supplementary Figure 5. Effective population size histories estimated using the PSMC method with the *de novo* assembled reference genome for each tree of the four taxa. Source data are provided as a Source Data file.

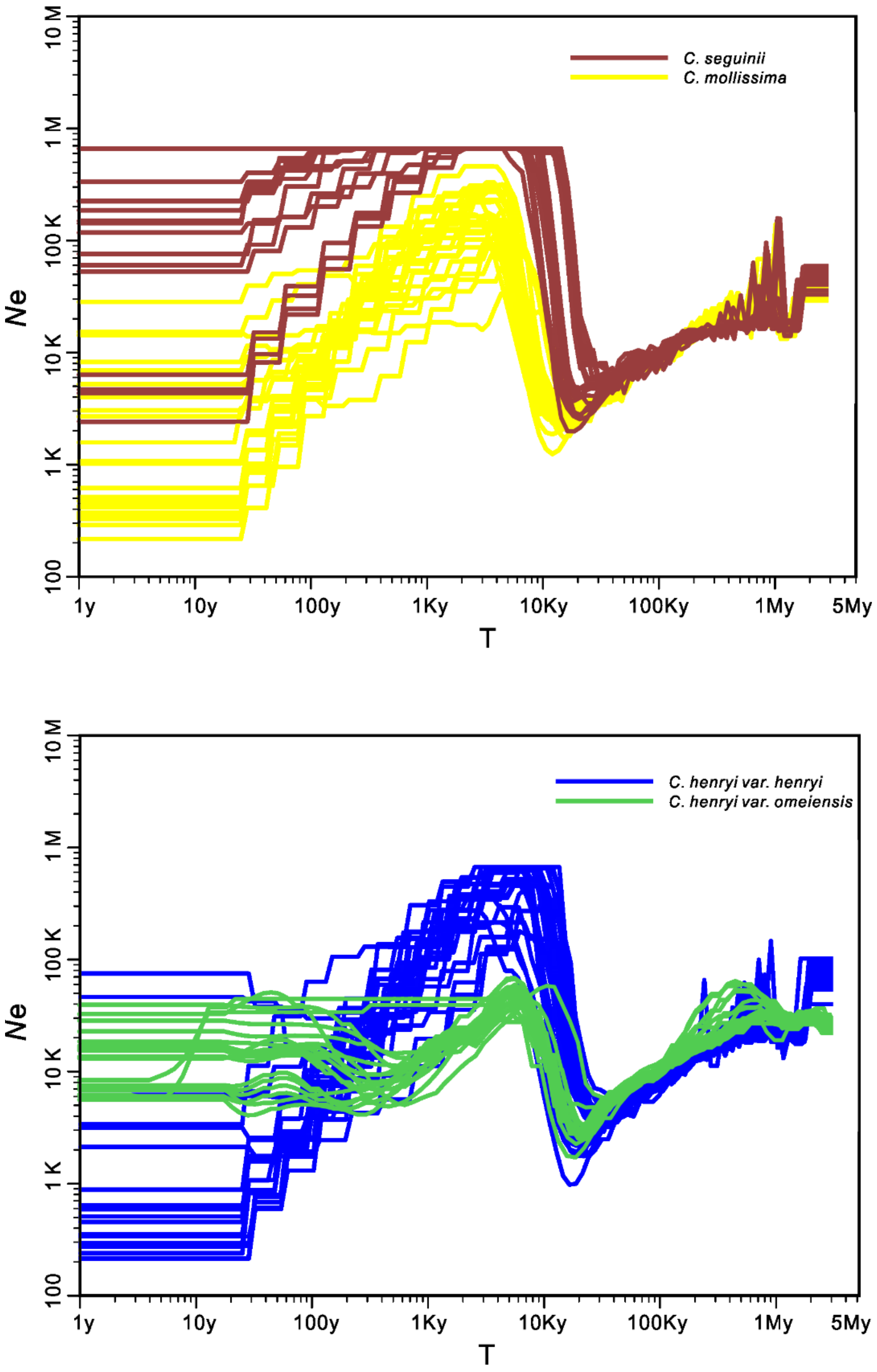

Supplementary Figure 6. Effective population size histories estimated using the SMC++ method with the genomic sequence for each tree of the four taxa. Source data are provided as a Source Data file.

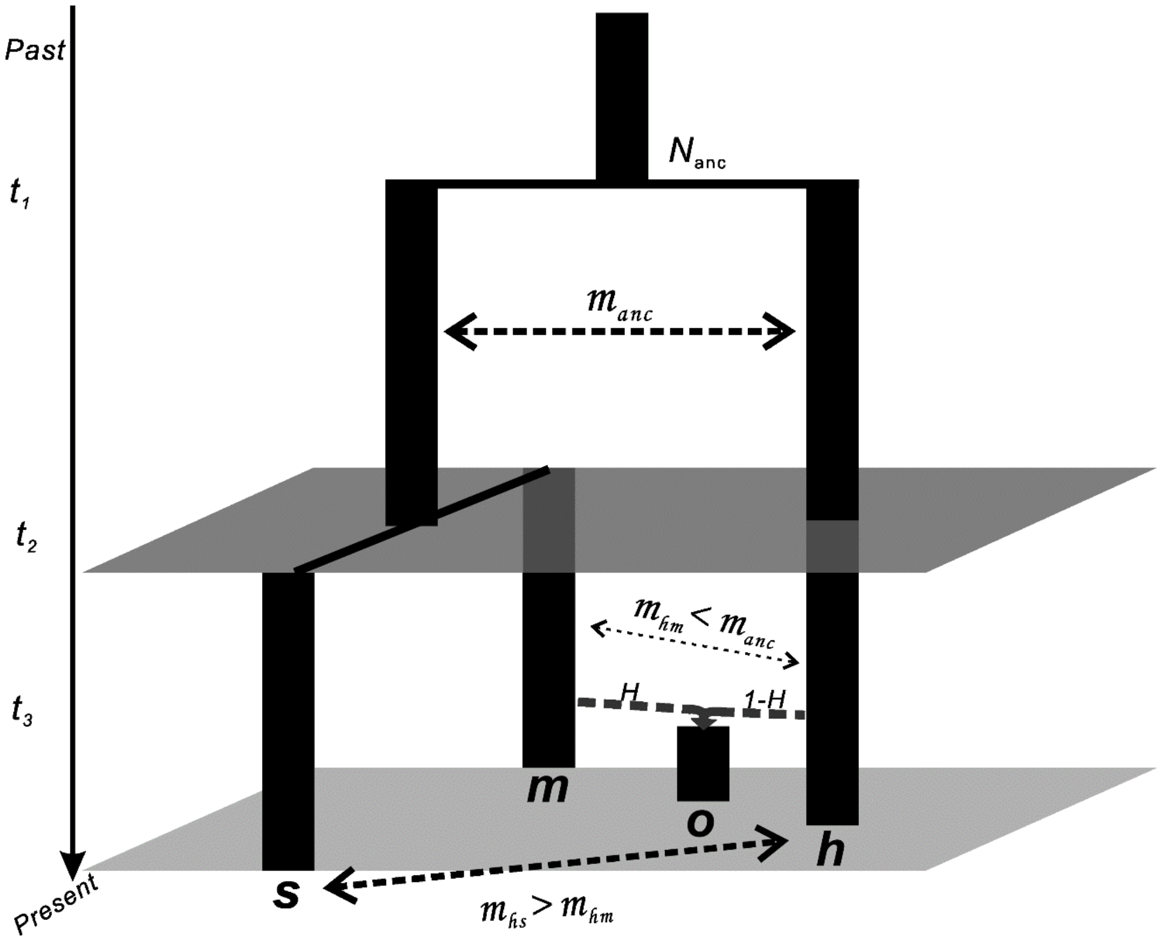

Supplementary Figure 7. The most likely evolutionary model used in the present study. Abbreviations are: s = *C. seguinii*, m = *C. mollissima*, h = *C. henryi* var. *henryi*, o = *C. henryi* var. *omeiensis*. At time t_1, h and the ancestral lineage of s and m diverged from an ancestral lineage with a population size of N_{anc}, which diverged from each other at time t_2. At time t_3, hybridization between m and h produced the hybrid lineage o. From t_1 to t_2, the two lineages exchanged alleles with migration rate m_{anc}. The parameters m_{hm} (m_{hs}) represent the migration rates between h and m (s) respectively after t_2. Source data are provided as a Source Data file.

Supplementary References

1 Tel-Zur, N., Abbo, S., Myslabodski, D. & Mizrahi, Y. Modified CTAB Procedure for DNA Isolation from Epiphytic Cacti of the Genera *Hylocereus* and *Selenicereus* (Cactaceae). *Plant Mol. Biol. Rep.* **17**, 249-254 (1999).

2 Petersen, K. R., Streett, D. A., Gerritsen, A. T., Hunter, S. S. & Settles, M. L. Super deduper, fast PCR duplicate detection in fastq files. *International conference on bioinformatics*, 491-492 (2015).

3 Bolger, A. M., Lohse, M. & Usadel, B. Trimmomatic: a flexible trimmer for Illumina sequence data. *Bioinformatics* **30**, 2114-2120 (2014).

4 Leggett, R. M., Clavijo, B. J., Leah, C., Clark, M. D. & Mario, C. NextClip: an analysis and read preparation tool for Nextera Long Mate Pair libraries. *Bioinformatics* **30**, 566-568 (2014).

5 Marçais, G. & Kingsford, C. A fast, lock-free approach for efficient parallel counting of occurrences of k-mers. *Bioinformatics* **27**, 764 (2011).

6 Langmead, B. & Salzberg, S. L. Fast gapped-read alignment with Bowtie 2. *Nat. Methods* **9**, 357-359 (2012).

7 Servant, N. *et al.* HiC-Pro: an optimized and flexible pipeline for Hi-C data processing. *Genome Biol.* **16**, 259-270 (2015).

8 Burton, J. N. *et al.* Chromosome-scale scaffolding of de novo genome assemblies based on chromatin interactions. *Nat. Biotechnol.* **31**, 1119-1125 (2013).

9 Tarailo-Graovac, M. & Chen, N. Using RepeatMasker to identify repetitive elements in genomic sequences. *Curr. Protoc. Bioinform.* **25**, 4.10. 11-14.10. 14 (2009).

10 Hubley, R. *et al.* The Dfam database of repetitive DNA families. *Nucleic Acids Res.* **44**, D81-D89 (2016).

11 Bao, W., Kojima, K. K. & Kohany, O. Repbase Update, a database of repetitive elements in eukaryotic genomes. *Mobile DNA* **6**, 11 (2015).

12 Xu, Z. & Wang, H. LTR_FINDER: an efficient tool for the prediction of full-length LTR retrotransposons. *Nucleic Acids Res.* **35**, W265-W268 (2007).

13 Ellinghaus, D., Kurtz, S. & Willhoeft, U. LTRharvest, an efficient and flexible software for de novo detection of LTR retrotransposons. *BMC Bioinformatics* **9**, 18 (2008).

14 Ou, S. & Jiang, N. LTR_retriever: a highly accurate and sensitive program for identification of long terminal repeat retrotransposons. *Plant Physiol.* **176**, 1410-1422 (2018).

15 Quinlan, A. R. BEDTools: the Swiss-army tool for genome feature analysis. *Curr. Protoc. Bioinform.* **47**, 11.12. 11-11.12. 34 (2014).

16 Stanke, M., Diekhans, M., Baertsch, R. & Haussler, D. Using native and syntenically mapped cDNA alignments to improve de novo gene finding. *Bioinformatics* **24**, 637-644 (2008).

17 Stanke, M. *et al.* AUGUSTUS: ab initio prediction of alternative transcripts. *Nucleic Acids Res.* **34**, W435-W439 (2006).

18 Majoros, W. H., Pertea, M. & Salzberg, S. L. TigrScan and GlimmerHMM: two open source ab initio eukaryotic gene-finders. *Bioinformatics* **20**, 2878-2879 (2004).

19 Lomsadze, A., Burns, P. D. & Borodovsky, M. Integration of mapped RNA-Seq reads into automatic training of eukaryotic gene finding algorithm. *Nucleic Acids Res.* **42**, e119-e119 (2014).

20 Keilwagen, J., Hartung, F. & Grau, J. in *Gene Prediction* (ed Kollmar M.) 161-177 (Springer, 2019).

21 Haas, B. J. *et al.* De novo transcript sequence reconstruction from RNA-seq using the Trinity platform for reference generation and analysis. *Nat. Protoc.* **8**, 1494-1512 (2013).

22 Gremme, G., Brendel, V., Sparks, M. E. & Kurtz, S. Engineering a software tool for gene structure prediction in higher organisms. *Inform. Software Tech.* **47**, 965-978 (2005).

23 Kim, D., Paggi, J. M., Park, C., Bennett, C. & Salzberg, S. L. Graph-based genome alignment and genotyping with HISAT2 and HISAT-genotype. *Nat. Biotechnol.* **37**, 907-915 (2019).

24 Haas, B. J. *et al.* Automated eukaryotic gene structure annotation using EVidenceModeler and the Program to Assemble Spliced Alignments. *Genome Biol.* **9**, R7 (2008).

25 Xing, Y. *et al.* Hybrid de novo genome assembly of Chinese chestnut (*Castanea mollissima*). *GigaScience* **8**, giz112 (2019).

26 Chan, A. H., Jenkins, P. A. & Song, Y., S. Genome-wide fine-scale recombination rate variation in *Drosophila melanogaster*. *PLoS Genet.* **8**, e1003090 (2012).
