# Supplementary material for: Genomic basis of homoploid hybrid speciation within chestnut trees
Source: Nat Commun. 2020 Jul 6;11:3375. doi: 10.1038/s41467-020-17111-w (PMC7338469; doi:10.1038/s41467-020-17111-w)
Supplement: Supplementary file 2 — Reporting Summary [file 41467_2020_17111_MOESM2_ESM.pdf]

nature research

Corresponding author(s): Yongshuai Sun

Last updated by author(s): Jun 5, 2020

Reporting Summary

Nature Research wishes to improve the reproducibility of the work that we publish. This form provides structure for consistency and transparency in reporting. For further information on Nature Research policies, see Authors & Referees and the Editorial Policy Checklist .

Statistics

For all statistical analyses, confirm that the following items are present in the figure legend, table legend, main text, or Methods section.

n/a	Confirmed	
☐	☒	The exact sample size (n) for each experimental group/condition, given as a discrete number and unit of measurement
☐	☒	A statement on whether measurements were taken from distinct samples or whether the same sample was measured repeatedly
☐	☒	The statistical test(s) used AND whether they are one- or two-sided<br>*Only common tests should be described solely by name; describe more complex techniques in the Methods section.*
☒	☐	A description of all covariates tested
☐	☒	A description of any assumptions or corrections, such as tests of normality and adjustment for multiple comparisons
☐	☒	A full description of the statistical parameters including central tendency (e.g. means) or other basic estimates (e.g. regression coefficient) AND variation (e.g. standard deviation) or associated estimates of uncertainty (e.g. confidence intervals)
☐	☒	For null hypothesis testing, the test statistic (e.g. F, t, r) with confidence intervals, effect sizes, degrees of freedom and P value noted<br>*Give P values as exact values whenever suitable.*
☐	☒	For Bayesian analysis, information on the choice of priors and Markov chain Monte Carlo settings
☒	☐	For hierarchical and complex designs, identification of the appropriate level for tests and full reporting of outcomes
☐	☒	Estimates of effect sizes (e.g. Cohen's d, Pearson's r), indicating how they were calculated

Our web collection on statistics for biologists contains articles on many of the points above.

Software and code

Policy information about availability of computer code

Data collection	Reference genome - Sequence data was collected by nanopore, Illumina and HiC with read calls produced by the provider platform software. Re-sequencing data was collected by Illumina instruments with read calls produced by the provider platform software.
Data analysis	Software packages: Super-Deduper(https://github.com/dstreett/Super-Deduper); Trimmomatic V0.35; NextClip V1.3.1; Jellyfish v2.2; SMARTdenovo(https://github.com/ruanjue/smartdenovo); WTDBG(https://github.com/ruanjue/wtdbg); NextDenovo (https://github.com/Nextomics/NextDenovo); NextPolish (https://github.com/Nextomics/NextPolish); BUSCO V3.0.1; HiC-Pro(https://github.com/nservant/HiC-Pro); bowtie2; LACHESIS(https://github.com/shendurelab/LACHESIS); RepeatModeler v2.0; RepeatMasker v4.0; LTR_retriever v2.8.6; bedtools v2.28; Augustus v3.2; Snap (http://korflab.ucdavis.edu/); GlimmerHMM v3; GeneMark-ET version 4.48_3.60_lic; GeMoMa 1.6.1; Trinity v2.8.5; PASA 2.4.0; GenomeThreader26; Hisat2; EVM v1.1; Interproscan5.24; KAAS(http://www.genome.jp/tools/kaas); FASTX-toolkit(http://hannonlab.cshl.edu/fastx_toolkit); BWA-MEM v0.7.16a-r1181; Picard-tools v1.92; samtools v12; GATK v4.0.8.1; ANGSD v0.928; PSMC(https://github.com/lh3/psmc); SMC++(https://github.com/popgenmethods/smcpp); fastsimcoal2; ADMIXTURE v1.23; FASTSTRUCTURE(https://github.com/rajanil/fastStructure); Python3 scripts (https://github.com/yongshuai-sun/hhs-omei) and R v13.0; LDhelmet v1.9; LDpop(https://github.com/popgenmethods/ldpop); HyDe(https://github.com/pblischak/HyDe).

For manuscripts utilizing custom algorithms or software that are central to the research but not yet described in published literature, software must be made available to editors/reviewers. We strongly encourage code deposition in a community repository (e.g. GitHub). See the Nature Research guidelines for submitting code & software for further information.

Data

Policy information about availability of data

All manuscripts must include a data availability statement. This statement should provide the following information, where applicable:

- Accession codes, unique identifiers, or web links for publicly available datasets
- A list of figures that have associated raw data
- A description of any restrictions on data availability

Dfam, Repbase and RepeatPeps, FLOR-ID, SwissProt, TrEMBL databases. Accesion code (Bioproject): PRJNA540917, https://dataview.ncbi.nlm.nih.gov/object/PRJNA540917?reviewer=e5mrafdh3dvpm2i3lccfivg91o . See manuscript figures. See source data online (https://github.com/yongshuai-sun/hhs-omei).

Field-specific reporting

Please select the one below that is the best fit for your research. If you are not sure, read the appropriate sections before making your selection.

☐ Life sciences ☐ Behavioural & social sciences ☒ Ecological, evolutionary & environmental sciences

For a reference copy of the document with all sections, see nature.com/documents/nr-reporting-summary-flat.pdf

Ecological, evolutionary & environmental sciences study design

All studies must disclose on these points even when the disclosure is negative.

Study description	The paper reports a case of homoploid hybrid speciation and describes fundamental discoveries about the role of natural selection and recombination in hybrid speciation of one Castanea taxon endemic in Mount Emei. In this hybrid system, two parental lineages have similar geographic distributions but distinct phenotypes. A third species, sister to one of the two parental species, provides an ideal phylogenetic control for identifying potential genes contributing to reproductive isolation. Furthermore, there are several unique aspects to our work. First, based on the nanopore sequencing data and Hi-C data, we provide a chromosome-scale genome of the Chinese chestnut, providing a solid basis for genomic analysis. Second, we use a large-scale population resequencing study to characterize polymorphisms across the range of four Castanea taxa. Third, we have used multiple methods to test the hypothesis of homoploid hybrid speciation. For the first time, we identify the barrier genomic regions isolating parental lineages, taking advantage of the phylogenetic control to exclude effects of random drift. Finally, we analyze the relationship between barrier loci and recombination rates.
Research sample	We generate a genome assembly for C. mollissima and genomic sequences of 115 chestnut trees. Most genetic variation can be captured when sample size is >=20 in traditional population genetics (see the book Mathematical Population Genetics by Warren J. Ewens in 1979). In our design, each lineage is treated as a population. So, we collected young leaves from 20 trees of C. henryi var. omeiensis, 24 trees of C. henryi var. henryi, 43 trees of C. mollissima and 28 trees of C. seguinii, spanning the geographic ranges of the four taxa (Figure 1 and Supplementary Tables 6-9), to represent the focused four populations.
Sampling strategy	The Chinese chestnut trees are important economic trees in China and are planted widely. We filtered out populations <50 km far from villages, cities and man-made chestnut forests, aiming to deduce effects of the domesticated Chinese chestnut trees. For each lineage, we sampled 20 or more trees to capture their genetic variation. We don't perform sample size assessment, because this sample size is thought to be suitable (see the book Mathematical Population Genetics by Warren J. Ewens in 1979).
Data collection	We used GATK4 and ANGSD to generate the dataset used in population genomics. This work was carried out by S. Y. and M. H.
Timing and spatial scale	In 2017-18, we collected leaves of 115 chestnut trees spanning their geographic ranges in China.
Data exclusions	No data were excluded, all genomes of 115 trees were used in this study. Bases with Phred quality score ≤ 20 were defined as low quality, because the accuracies of these bases are lower than 99%. Low quality bases were masked, and were trimmed if they were at end of the read.
Reproducibility	Four trees of C. mollissima and three trees of C. henryi were used to assess the quality of the SNPs called by the GATK4-pipeline above, based on duplicated re-sequencing. For each of 7 trees, two separate DNA samples were sequenced. These datasets were processed in identical pipeline with the processing from quality control to the GATK4-pipeline, blind to the fact that they were duplicates. Then we compared the genotypes in the two duplicated samples for each of 7 trees. Further, we verified the SNPs using population re-sequenced data of C. mollissima and C. henryi. The rationale is that, if a heterozygotic genotype from one tree genome cannot be detected in its duplicated sequencing data or be found in population samples, it would be counted as an unverified genotype. The maximum proportion of unverified heterozygotic genotypes for each tree is 0.000167%, corresponding to a Phred-scaled score of 57.8, suggesting that variants detected by the present GATK4-pipeline is of high quality.
Randomization	All 115 samples were allocated into one of three species (C. mollissima, C. seguinii, C. henryi) when collecting the leaves, according to their distinct morphological traits. To assure the correctness, we performed genetic delimitation using clustering analyses, including ADMIXTURE, FASTSTRUCTURE.
Blinding	When collecting samples and analyzing data, the analyst knew nothing about the demography of each species, their relationships and whether there is hybridization among them. When estimating SNPs, we used a double blind experiment to validate the quality of our SNPs data.

Did the study involve field work? ☐ Yes ☒ No

Reporting for specific materials, systems and methods

We require information from authors about some types of materials, experimental systems and methods used in many studies. Here, indicate whether each material, system or method listed is relevant to your study. If you are not sure if a list item applies to your research, read the appropriate section before selecting a response.

Materials & experimental systems

n/a	Involved in the study
☒	☐ Antibodies
☒	☐ Eukaryotic cell lines
☒	☐ Palaeontology
☒	☐ Animals and other organisms
☒	☐ Human research participants
☒	☐ Clinical data

Methods

n/a	Involved in the study
☒	☐ ChIP-seq
☒	☐ Flow cytometry
☒	☐ MRI-based neuroimaging
